# Exercise-responsive microRNA networks and extracellular vesicle–mediated microRNA signaling in breast cancer: linking tumor signaling, systemic crosstalk, and clinical relevance

**DOI:** 10.3389/fonc.2026.1849217

**Published:** 2026-06-18

**Authors:** Qi Ye, Shoudu Yuan, Duanhao Wang

**Affiliations:** 1 Physical Education Institute, Chongqing College of Humanities, Science and Technology, Chongqing, China; 2 School of Physical Education and Health, Chongqing College of International Business and Economics, Chongqing, China; 3 Chongqing Institute of Sport Science, Chongqing, China

**Keywords:** breast cancer, circulating biomarkers, exercise, exercise oncology, extracellular vesicles, microRNAs, tumor microenvironment

## Abstract

Breast cancer is increasingly recognized as a systemic disease shaped by dynamic interactions between tumor-intrinsic signaling and host physiology. MicroRNAs (miRNAs), as post-transcriptional regulators, extend beyond canonical gene silencing to coordinate oncogenic pathways, tumor microenvironment remodeling, and inter-organ communication. In parallel, exercise has emerged as a systemic modulator capable of influencing immune, metabolic, and circulatory processes relevant to tumor progression. This review integrates current evidence on the interplay between miRNAs and exercise in breast cancer. We examine how miRNA-mediated networks regulate key processes including oncogenic signaling, angiogenesis, hypoxia responses, immune modulation, and metabolic adaptation. Particular attention is given to circulating and extracellular vesicle-associated miRNAs as mediators of systemic signaling, including muscle–tumor crosstalk. Emerging clinical data further support the role of circulating miRNAs as minimally invasive biomarkers for early detection and diagnosis, risk stratification, and monitoring of treatment response, with growing relevance to physical activity, overall health status, and lifestyle-based interventions that integrate exercise and behavioral modification strategies. Overall, this review proposes a systems-oriented framework in which miRNAs may link exercise-induced physiological adaptation to breast cancer biology, providing a foundation for future translational and precision oncology strategies.

## 1 Introduction

Breast cancer represents a biologically complex and heterogeneous disease entity, the progression of which cannot be fully explained by tumor-intrinsic mechanisms alone. Although decades of research have delineated key genetic alterations and signaling pathways that drive tumor initiation and growth, these insights provide only a partial view of disease dynamics (1). Increasing evidence suggests that breast cancer behavior is shaped not only by the molecular characteristics of malignant cells, but also by continuous and dynamic interactions with the host organism. These interactions extend beyond the immediate tumor microenvironment and involve systemic physiological processes that influence tumor evolution, therapeutic responsiveness, and disease outcomes. Consequently, there is a growing recognition that a tumor-centered framework, while informative, may be insufficient to fully capture the multidimensional nature of breast cancer biology (2, 3). Within this broader context, regulatory molecules that can integrate signals across multiple biological levels have attracted considerable attention. Among these, microRNAs (miRNAs) have emerged as key post-transcriptional regulators capable of modulating gene expression programs in a coordinated and context-dependent manner (4). By targeting multiple transcripts simultaneously, miRNAs contribute to the fine regulation of cellular processes such as proliferation, differentiation, stress response, and phenotypic plasticity.

Beyond their intracellular functions, the detection of miRNAs in circulation has further expanded their relevance, suggesting that they may reflect systemic physiological states as well as local tumor-associated processes (5, 6). This dual role positions miRNAs at the interface between molecular regulation and organism-level signaling, highlighting their potential importance in shaping cancer biology across multiple scales (7). In parallel, a substantial body of research has demonstrated that systemic physiological factors can influence cancer progression in meaningful ways. Among these, physical activity has consistently been associated with improved clinical outcomes in patients with breast cancer, including reduced recurrence risk and enhanced survival. These observations have traditionally been interpreted within a supportive care framework; however, emerging findings indicate that exercise may exert broader biological effects that extend beyond general health benefits (8–10). Exercise-induced adaptations in immune function, metabolic regulation, and vascular dynamics have been proposed as potential mechanisms through which systemic physiology can influence tumor behavior. Despite these advances, the precise biological pathways linking systemic adaptations to molecular regulatory processes remain incompletely characterized (11, 12). Notwithstanding the growing interest in both miRNA biology and systemic modulation of cancer, current knowledge in these domains remains largely compartmentalized. Investigations of miRNA function have predominantly focused on their roles within tumor cells or specific signaling pathways, often without considering how systemic physiological conditions may shape their expression and activity. Conversely, studies examining the impact of exercise and other systemic factors have tended to emphasize clinical or organism-level outcomes, with comparatively limited integration of underlying molecular regulatory networks (13, 14). This separation has resulted in a conceptual gap, whereby the potential interactions between systemic physiological adaptation and miRNA-mediated regulation are not fully understood within a unified framework.

Addressing this gap requires a shift toward a more integrative perspective that considers cancer progression as the outcome of multilayered regulatory interactions spanning molecular, cellular, and systemic domains (15). Within such a framework, miRNAs can be viewed not only as intracellular regulators but also as dynamic components of broader communication networks that reflect and potentially mediate organism-level changes (16). At the same time, systemic modulators such as exercise may influence tumor biology indirectly by reshaping the physiological environment in which cancer develops, thereby altering the regulatory landscape in which molecular processes operate (17). The convergence of these concepts suggests the need for a systems-level approach that bridges molecular mechanisms with whole-body physiology. The present review adopts this integrative perspective to explore the interplay between miRNA-mediated regulation and systemic physiological processes in breast cancer. Rather than treating these elements as independent contributors, this work examines how they may function in a coordinated manner to influence disease progression and therapeutic response. Particular emphasis is placed on the role of systemic modulators in shaping the biological context of tumor development, and on the potential of miRNAs to serve as both mediators and indicators of these processes. By situating molecular regulation within a broader physiological framework, the review aims to provide a more cohesive understanding of breast cancer biology. Importantly, this approach does not seek to replace established mechanistic insights, but to contextualize them within a more comprehensive and interconnected model. Integrating knowledge across different levels of biological organization may facilitate the identification of previously unrecognized relationships between systemic physiology and tumor behavior. Such an understanding has the potential to inform future research directions and support the development of more nuanced strategies for cancer management. Collectively, this work contributes to an evolving paradigm in which breast cancer is viewed as a disease shaped by continuous interactions between tumor cells and the host system, underscoring the importance of considering both local and systemic determinants in advancing cancer research.

## 2 MicroRNA regulatory networks in breast cancer: from canonical gene silencing to systems-level signaling integration

miRNAs are endogenous non-coding RNA molecules that play a central role in the post-transcriptional regulation of gene expression. Although they predominantly bind to partially complementary sequences within the 3′ untranslated regions (3′UTRs) of target messenger RNAs, binding to the 5′ untranslated region (5′UTR) and coding sequences has also been reported. Depending on the degree of complementarity—ranging from near-perfect to partial—miRNA–mRNA interactions can lead to different outcomes, including translational repression, mRNA cleavage, or transcript destabilization. This mode of action enables a single miRNA to regulate multiple genes simultaneously, while individual transcripts may be targeted by several miRNAs, giving rise to a densely interconnected regulatory architecture. Such many-to-many interactions distinguish miRNA-mediated regulation from linear gene control mechanisms and position miRNAs as key modulators of cellular homeostasis (18, 19). A defining feature of miRNA function is its capacity to fine-tune gene expression in a graded and context-sensitive manner, rather than enforcing absolute on–off responses. This property allows cells to maintain functional stability under physiological conditions while retaining the flexibility to adapt to environmental or intracellular perturbations (20). In pathological settings, including cancer, this regulatory plasticity becomes particularly relevant, as it enables dynamic shifts in cellular behavior. Processes such as proliferation, invasion, and resistance to therapy are increasingly understood to emerge from coordinated regulatory adjustments rather than isolated molecular events, with miRNAs contributing significantly to this adaptive capacity (21).

Beyond this functional flexibility, accumulating evidence indicates that miRNAs operate within multilayered regulatory networks that extend far beyond one-to-one gene interactions. Rather than acting as isolated molecular switches, they serve as integrative regulators that coordinate gene expression across multiple signaling pathways simultaneously. Their regulatory effects are inherently context-dependent and can vary according to cellular phenotype, tumor subtype, microenvironmental conditions, and disease stage. This systems-level behavior implies that the biological impact of a given miRNA cannot be fully interpreted through individual targets alone, but must instead be understood within the broader network in which it operates (22, 23). Within the field of breast cancer research, early studies largely interpreted miRNA function through a simplified dichotomous model, categorizing them as either oncogenic miRNAs (oncomiRs) or tumor-suppressive miRNAs. OncomiRs are typically upregulated in malignant tissues and contribute to tumorigenesis by suppressing genes involved in apoptosis, cell cycle control, and differentiation. In contrast, tumor-suppressive miRNAs are often downregulated, leading to the derepression of oncogenic signaling pathways. While this classification provided a useful conceptual framework, it is now recognized as an oversimplification that does not fully capture the complexity of miRNA-mediated regulation in cancer. Increasing evidence suggests that miRNAs can exhibit context-dependent roles, with their functional effects influenced by the broader regulatory environment in which they are embedded (24, 25).

Extending beyond intracellular regulation, miRNAs have also been implicated in mediating communication between cells and across tissues. Circulating miRNAs, detectable in biological fluids such as serum, plasma, and urine, display notable stability due to their association with RNA-binding proteins or encapsulation within extracellular vesicles. This stability has driven considerable interest in their application as minimally invasive biomarkers. At the same time, their presence in circulation suggests a broader role in systemic signaling, where miRNAs may participate in the transfer of molecular information between tumors and distant organs, thereby contributing to disease progression at the organismal level (26, 27). Collectively, these insights have reshaped the understanding of miRNAs from simple post-transcriptional regulators to dynamic components of complex biological systems. In breast cancer, where disease progression is governed by the interplay of multiple signaling pathways and environmental influences, miRNAs can be viewed as critical nodes that integrate molecular regulation with broader cellular and systemic processes. This evolving perspective underscores the importance of examining miRNA function within interconnected regulatory frameworks, providing a conceptual basis for exploring their roles in more complex, multilayered contexts.

## 3 Breast cancer as a systemic disease: integrating immune, metabolic, and inter-organ regulatory networks

Breast cancer has traditionally been conceptualized as a localized disease originating within mammary tissue and progressing through well-defined stages of tumor growth and metastatic dissemination. This tumor-centric perspective has historically guided both mechanistic investigations and therapeutic strategies, with primary emphasis placed on intrinsic properties of cancer cells, such as genetic mutations, proliferative capacity, and invasive behavior. While this framework has yielded critical insights into tumor biology, it is increasingly evident that it does not fully capture the complexity of breast cancer progression (28, 29). Emerging evidence supports a more integrated view in which breast cancer is understood as a systemic disease shaped by continuous interactions between the tumor and the host organism. Within this broader context, tumor development and progression are not solely determined by cell-autonomous mechanisms but are profoundly influenced by systemic physiological processes, including immune regulation, metabolic homeostasis, and endocrine signaling. These factors collectively contribute to a dynamic biological environment that can either constrain or facilitate tumor growth and dissemination (30, 31). The immune system represents one of the most influential systemic components in this interplay. Rather than functioning as a static defense mechanism, immune responses are highly adaptable and can exert both tumor-suppressive and tumor-promoting effects depending on context. The balance between cytotoxic immune activity and immunosuppressive signaling within the host plays a critical role in determining disease trajectory. Importantly, systemic immune modulation can alter not only local tumor microenvironments but also distant tissue niches that may support metastatic colonization. This highlights the need to consider cancer progression within the broader framework of host immune dynamics (32, 33). In parallel, metabolic regulation has emerged as a key determinant of cancer behavior at the organismal level. Tumor cells exhibit metabolic flexibility that enables them to adapt to fluctuating nutrient availability and energetic demands.

However, these adaptations are not confined to the tumor itself; they are closely linked to systemic metabolic states, including insulin sensitivity, lipid metabolism, and overall energy balance. Alterations in these systemic parameters can influence tumor growth indirectly by modifying the availability of metabolic substrates, as well as directly by affecting signaling pathways that regulate proliferation and survival. Consequently, metabolic status should be viewed as an integral component of cancer biology rather than a peripheral factor (34, 35). Beyond immune and metabolic influences, endocrine and circulating signaling factors further contribute to the systemic nature of breast cancer. Hormones, cytokines, and other circulating mediators form a complex communication network that connects distant tissues with the tumor. These signals can modulate tumor cell behavior, reshape the tumor microenvironment, and influence therapeutic responsiveness. Importantly, such systemic communication is bidirectional: tumors can actively alter host physiology, leading to widespread changes that extend beyond the primary disease site (36, 37). This expanded perspective underscores that breast cancer progression arises from the integration of local and systemic processes rather than from tumor-intrinsic mechanisms alone. It also highlights the importance of considering inter-organ communication as a fundamental component of disease biology. In this context, peripheral tissues—particularly metabolically active organs such as skeletal muscle—may play underappreciated roles in modulating tumor behavior through the release of circulating factors and regulatory molecules (38, 39). A systems-level perspective reframes breast cancer as a disease influenced by organism-wide physiological states rather than tumor-intrinsic factors alone. Modulation of systemic processes, including physical activity and metabolic balance, may alter tumor behavior indirectly by reshaping the host environment, highlighting the importance of integrating whole-body adaptations into cancer biology.

## 4 Exercise as a systemic modulator in cancer biology: integrating immune, metabolic, and circulatory dynamics

Building on the previously discussed molecular and cellular mechanisms, it is important to consider how these effects are integrated at the organismal level. Exercise influences multiple physiological networks simultaneously, including immune surveillance, metabolic regulation, and circulatory function, thereby contributing to an internal environment that is less favorable for tumor progression and more supportive of anti-cancer defense mechanisms. While exercise has traditionally been considered a supportive lifestyle intervention in cancer care, primarily improving quality of life, physical function, and treatment tolerance (40), accumulating evidence indicates that its biological impact extends beyond symptomatic benefits. Importantly, exercise can be viewed as a multi-level regulator of host physiology that affects interconnected pathways involved in cancer development and progression (41). Within this systemic framework, one of the most extensively studied dimensions is its effect on immune function. Physical activity has been shown to influence both innate and adaptive immune responses, altering the distribution, activation state, and functional capacity of key immune cell populations (42). These changes are not limited to transient fluctuations but may contribute to sustained shifts in immune surveillance and inflammatory balance. Exercise-associated modulation of immune dynamics has been linked to enhanced cytotoxic activity, improved antigen recognition, and a reduction in chronic low-grade inflammation. Such systemic immune adaptations have the potential to reshape the host environment in ways that may constrain tumor growth and limit disease progression (43, 44). In addition to immune regulation, exercise plays a critical role in shaping systemic metabolic homeostasis. Cancer is closely associated with metabolic dysregulation, including altered glucose utilization, lipid metabolism, and energy balance (45).

Regular physical activity contributes to improved metabolic efficiency by enhancing insulin sensitivity, promoting mitochondrial function, and regulating substrate utilization across multiple tissues. These systemic metabolic adjustments can influence tumor behavior indirectly by modifying the availability of nutrients and metabolic intermediates required for cancer cell proliferation (11). Furthermore, exercise-induced metabolic reprogramming may affect signaling pathways that link energy status to cellular growth and survival, thereby contributing to a less favorable environment for tumor progression (46, 47). Another important aspect of exercise-mediated systemic regulation involves its effects on circulation and vascular function. Exercise induces widespread hemodynamic changes, including increased blood flow, improved endothelial function, and enhanced tissue perfusion. These adaptations can influence the distribution of oxygen, nutrients, and signaling molecules throughout the body. Improved vascular function may also facilitate more efficient delivery of therapeutic agents and immune cells to target tissues. At the same time, exercise-associated changes in circulation may contribute to the normalization of aberrant vascular structures commonly observed in tumors, thereby altering the physiological conditions within the tumor microenvironment (48–50). Importantly, the biological effects of exercise are inherently dose-dependent and context-specific. Variations in intensity, duration, and frequency of physical activity can lead to distinct physiological responses, with potentially divergent implications for cancer outcomes. While moderate levels of exercise are generally associated with beneficial systemic adaptations, excessive or insufficient activity may not confer the same advantages. This highlights the importance of considering exercise as a modifiable and precisely tunable intervention rather than a uniform exposure (51, 52). Taken together, these observations support a conceptual shift in which exercise is viewed not merely as an adjunctive lifestyle factor, but as a systemic modulator that influences the complex interplay between host physiology and tumor biology. By simultaneously impacting immune regulation, metabolic balance, and circulatory dynamics, exercise contributes to the reconfiguration of the biological environment in which cancer evolves (53, 54). This integrative perspective provides a foundation for understanding how organism-level interventions may shape disease trajectories and underscores the potential of exercise-informed strategies in cancer research and management.

## 5 MicroRNA-driven multilayered regulation of breast cancer progression: from oncogenic signaling to tumor microenvironment and systemic remodeling

Regulatory complexity in cancer is increasingly understood as the result of coordinated interactions across multiple biological layers rather than isolated molecular events. Within this framework, miRNAs act as critical integrators that modulate gene expression programs linking intracellular signaling with microenvironmental and systemic dynamics (55). Emerging evidence indicates that miRNA-mediated networks simultaneously influence oncogenic pathways, vascular adaptation, and immune–metabolic interactions. In this section, we examine these roles across three interconnected dimensions, focusing on how miRNAs contribute to signaling reprogramming, angiogenesis and hypoxia responses, and immune–metabolic–epigenetic regulation in breast cancer.

### 5.1 MiRNA-mediated reprogramming of core oncogenic signaling in breast cancer progression and metastasis

Breast cancer development and progression are governed by a complex network of oncogenic signaling pathways that regulate essential cellular processes, including proliferation, survival, migration, invasion, angiogenesis, and therapeutic responsiveness. Central signaling axes such as Phosphoinositide 3-kinase/Protein kinase B (PI3K/Akt), Wnt/β-catenin, Nuclear factor kappa B (NF-κB), and Nuclear factor kappa B (TGF-β) do not function independently but rather form an interconnected regulatory framework that drives tumor evolution and metastatic spread (56). Increasing evidence suggests that miRNAs operate as key upstream modulators of these pathways, fine-tuning gene expression programs that ultimately shape tumor behavior. To provide a concise overview of these multilayered regulatory interactions, key microRNAs modulating core oncogenic signaling pathways in breast cancer are summarized in **Table 1**. Depending on their targets, miRNAs can function either as oncogenic regulators or tumor suppressors, thereby influencing diverse processes such as epithelial–mesenchymal transition (EMT), resistance to therapy, and maintenance of cancer stem cell populations (57). As a result, miRNA-mediated control of oncogenic signaling represents a fundamental mechanistic layer in breast cancer biology. One of the most clinically significant features of breast cancer is the development of resistance to endocrine and chemotherapeutic treatments, a multifactorial process in which aberrant miRNA expression has been frequently implicated, although it is not considered the sole determining factor for therapy resistance or loss of hormonal markers. For example, miR-221/222 has been identified as a critical mediator of resistance to fulvestrant in estrogen receptor-positive breast cancer. Elevated expression of these miRNAs enables tumor cells to bypass hormone dependency, sustain proliferation, and maintain cell cycle progression under anti-estrogen conditions (58). Mechanistically, miR-221/222 activates β-catenin signaling while concurrently attenuating TGF-β-mediated growth suppression, thereby promoting a shift toward a more aggressive and therapy-resistant phenotype (58).

**Table 1:** Key microRNAs regulating core oncogenic signaling pathways in breast cancer progression and metastasis.

miRNA	Regulation (Up/Down)	Target gene(s)	Pathway	Functional outcome	Reference
miR-221/222	Up	β-catenin regulators; TGF-β components	Wnt/β-catenin; TGF-β	Endocrine resistance, sustained proliferation	(58)
miR-130b	Up	PTEN	PI3K/Akt	Multidrug resistance, reduced apoptosis	(59)
miR-3646	Up	GSK-3β	Wnt/β-catenin	Chemoresistance, enhanced survival	(60)
miR-133a	Down	EGFR	PI3K/Akt	Inhibition of proliferation, cell cycle arrest	(61)
miR-147	Up	Akt/mTOR components	PI3K/Akt/mTOR	Suppressed proliferation and invasion	(62)
miR-590	Up	JAK2, PI3K, MAPK1, CREB	JAK/STAT; PI3K/Akt; MAPK	Enhanced apoptosis, reduced metastasis	(63)
miR-340	Down	CTNNB1, c-MYC, ROCK1	Wnt/β-catenin	Inhibition of metastasis and migration	(64)
miR-100	Up	FZD-8	Wnt/β-catenin	Reduced invasion and motility	(65)
miR-520/373	Up	RELA, TGFBR2	NF-κB; TGF-β	Suppression of inflammation and invasion	(66)
miR-200c	Down	BMI1	Stemness regulation	Reduced cancer stem cell maintenance	(67)

Wnt, wingless-related integration site; β-catenin, beta-catenin; TGF-β, transforming growth factor beta; PI3K, phosphoinositide 3-kinase; Akt, protein kinase B; mTOR, mechanistic target of rapamycin; JAK, Janus kinase; STAT, signal transducer and activator of transcription; MAPK, mitogen-activated protein kinase; CREB, cAMP response element-binding protein; NF-κB, nuclear factor kappa B.

In a similar manner, miR-130b contributes to multidrug resistance through direct targeting of Phosphatase and tensin homolog)PTEN(, resulting in activation of the PI3K/Akt pathway, enhanced cellular proliferation, and reduced apoptotic response (59). Furthermore, miR-3646 has been shown to promote resistance to docetaxel by modulating the Glycogen synthase kinase 3 beta (GSK-3β (/β-catenin signaling axis. Suppression of GSK-3β leads to accumulation of β-catenin, which in turn activates transcriptional programs associated with survival and drug resistance (60). Collectively, these findings highlight that miRNAs can rewire core oncogenic pathways to facilitate tumor adaptation under therapeutic pressure. In addition to their role in drug resistance, miRNAs exert substantial control over tumor cell proliferation by regulating receptor-mediated signaling pathways. MiR-133a, which is frequently downregulated in breast cancer, has been shown to suppress cell proliferation by directly targeting the epidermal growth factor receptor (EGFR) and inhibiting downstream Akt signaling. Restoration of miR-133a expression leads to cell cycle arrest and reduced DNA synthesis, indicating its function as a potent tumor suppressor (61). Similarly, miR-147 plays an inhibitory role in tumor progression by targeting the Akt/mTOR pathway, a central regulator of cellular growth and metabolism. Increased expression of miR-147 results in decreased phosphorylation of key signaling components, ultimately suppressing proliferation, invasion, and migration of breast cancer cells (62). In parallel, miR-590 has been shown to simultaneously downregulate multiple oncogenic signaling nodes, including Janus kinase 2 (JAK2), PI3K, Mitogen-activated protein kinase 1(MAPK1), and cAMP response element-binding protein (CREB), leading to enhanced apoptosis and reduced metastatic potential (63). These observations suggest that certain miRNAs function as broad-spectrum regulators capable of targeting multiple pathways, thereby exerting amplified anti-tumor effects.

Metastatic progression, which accounts for the majority of breast cancer-related deaths, is closely associated with the activation of signaling pathways that facilitate cell motility, invasion, and extracellular matrix remodeling. Among these, the Wnt/β-catenin pathway plays a pivotal role in driving metastatic behavior. MiR-340 has been identified as a strong suppressor of metastasis by targeting key components of both canonical and non-canonical Wnt signaling pathways, including Catenin beta 1 (CTNNB1), c-MYC, and Rho-associated coiled-coil containing protein kinase 1(ROCK1) (64). Restoration of miR-340 expression significantly reduces invasive and migratory capabilities in highly metastatic breast cancer cells, emphasizing its importance in controlling tumor dissemination (64). Likewise, miR-100 inhibits migration and invasion by targeting FZD-8, a critical receptor within the Wnt/β-catenin pathway, and subsequently downregulating downstream effectors such as β-catenin, MMP-7, TCF-4, and LEF-1. This coordinated suppression disrupts transcriptional programs required for metastatic progression (65). Additionally, the miR-520/373 family acts as a metastasis suppressor by targeting both NF-κB and TGF-β signaling pathways. By inhibiting RELA and TGFBR2, these miRNAs reduce inflammatory cytokine production and suppress invasion-associated gene expression, thereby limiting tumor dissemination (66). This dual regulatory role underscores the capacity of miRNAs to integrate multiple signaling pathways involved in metastasis and inflammation. Another critical component of tumor progression is the presence of cancer stem cells, which contribute to tumor initiation, heterogeneity, and resistance to therapy. The miR-200 family, particularly miR-200c, has been identified as a key regulator of CSC properties in breast cancer. Downregulation of miR-200c is associated with increased self-renewal capacity and expansion of CSC populations through modulation of BMI1, a central regulator of stem cell maintenance.

Restoration of miR-200c expression suppresses clonal expansion and tumor formation, indicating its essential role in controlling cellular plasticity and tumor hierarchy (67). These findings highlight the importance of miRNA-mediated regulation in maintaining the balance between differentiation and stemness within the tumor microenvironment. Importantly, the signaling pathways involved in tumor progression are highly interconnected, forming a dynamic and adaptive network. Crosstalk between pathways such as PI3K/Akt, Wnt/β-catenin, NF-κB, and TGF-β enables tumor cells to rapidly adjust to environmental changes and therapeutic interventions. For instance, activation of PI3K/Akt signaling can enhance β-catenin activity, while NF-κB signaling can cooperate with TGF-β pathways to promote inflammatory and metastatic responses. MiRNAs, by targeting multiple components within these pathways, serve as critical integrators that modulate the overall signaling landscape. This integrative function is particularly evident in miRNAs such as miR-520/373 and miR-590, which simultaneously regulate multiple signaling pathways involved in proliferation, inflammation, and metastasis (63, 66). Through such coordinated regulation, miRNAs fine-tune the balance between tumor-promoting and tumor-suppressive processes. From a translational perspective, targeting miRNA-mediated signaling networks offers a promising strategy for improving breast cancer treatment. Inhibition of oncogenic miRNAs such as miR-221/222 or miR-130b may restore sensitivity to endocrine and chemotherapeutic agents, while reintroduction of tumor-suppressive miRNAs such as miR-133a, miR-340, and miR-200c may inhibit proliferation, metastasis, and stemness. However, the context-dependent effects of miRNAs present a significant challenge, as their regulatory roles may vary across tumor subtypes and microenvironmental conditions. Therefore, a comprehensive understanding of miRNA–signaling interactions is essential for the development of effective and targeted therapeutic strategies. In conclusion, miRNAs play a central role in orchestrating core oncogenic signaling pathways that drive breast cancer progression, metastasis, and therapeutic resistance. By modulating key pathways such as PI3K/Akt, Wnt/β-catenin, NF-κB, and TGF-β, miRNAs act as critical regulators of tumor behavior. Their ability to simultaneously influence multiple signaling networks positions them as both biomarkers and potential therapeutic targets. Understanding these complex regulatory interactions provides a strong mechanistic foundation for future strategies aimed at improving clinical outcomes in breast cancer.

### 5.2 MicroRNA-mediated regulation of angiogenesis and hypoxia in breast cancer: implications for exercise-driven tumor microenvironment remodeling

Angiogenesis and hypoxia-responsive signaling are key components of tumor progression in breast cancer, primarily mediated through VEGF-centered pathways and upstream regulators such as Hypoxia-inducible factor 1 alpha (HIF-1α) and Signal transducer and activator of transcription 3 (STAT3). Increasing evidence indicates that miRNAs act as important modulators of these processes, influencing vascular formation, tumor invasion, and microenvironmental adaptation. However, the interplay between these molecular regulators and systemic interventions such as exercise remains incompletely understood (68, 69). A number of studies integrating exercise, miRNA regulation, and breast cancer provide initial mechanistic insights into how angiogenesis-related pathways may be modulated. In the study by Isanejad et al. (70), interval exercise training, particularly when combined with endocrine therapies, was associated with significant modulation of angiogenesis-related markers. They reported that exercise increased miR-206 and let-7 expression while reducing miR-21 levels, accompanied by decreased expression of VEGF, HIF-1α, CD31, and Ki67. These findings suggest that exercise may influence tumor vascularization through coordinated regulation of both pro- and anti-angiogenic miRNAs, although the exact causal pathways remain to be fully clarified. Similarly, Khori et al. (71) demonstrated that exercise training combined with tamoxifen reduced tumor burden and modulated angiogenesis-related signaling networks. Their findings showed that exercise led to downregulation of miR-21 and suppression of NF-κB and STAT3 pathways, alongside reductions in IL-6 and ER-α levels. Given the role of STAT3 and inflammatory mediators in promoting VEGF expression, these results suggest that exercise may indirectly influence angiogenesis through modulation of upstream signaling cascades. In another experimental study, Amani-Shalamzari et al. (72) reported that endurance training significantly reduced the expression of miR-21, STAT3, and B-cell lymphoma 2 (Bcl-2), which coincided with decreased tumor growth. They suggested that suppression of the STAT3/miR-21 axis may contribute to reduced angiogenic signaling, although they acknowledged that multiple interconnected pathways are likely involved. This highlights the complexity of interpreting exercise-induced molecular changes in the context of tumor biology. Further evidence is provided by Rafiei et al. (73), who investigated the effects of aerobic training on angiogenesis and apoptosis-related genes. They observed that exercise reduced tumor volume and miR-21 expression while modulating HIF-1α, VEGF, and apoptotic markers such as caspases. Although VEGF changes were not consistently significant, Rafiei et al. (73) suggested that exercise may alter the balance between angiogenesis and apoptosis within the tumor microenvironment, potentially contributing to slower tumor progression. In addition to these integrative studies, other experimental findings provide more direct insight into miRNA-mediated regulation of angiogenesis.

For instance, aerobic exercise has been associated with increased expression of miR-15a and reduced levels of HIF-1α and VEGF, suggesting a potential anti-angiogenic shift (74). This observation supports the idea that exercise may influence angiogenesis not only through suppression of pro-angiogenic pathways but also via activation of inhibitory miRNAs. Exercise intensity may further modulate these responses. High-intensity interval training (HIIT) has been shown to alter the expression of miRNAs such as miR-126 and miR-296, alongside reductions in VEGF-A protein levels. Given that these miRNAs are involved in endothelial function and vascular remodeling; their modulation may contribute to changes in tumor angiogenesis. However, the direction and magnitude of these effects appear to depend on experimental conditions, highlighting the need for cautious interpretation (75). Beyond exercise-related mechanisms, several miRNAs have been identified as direct regulators of angiogenesis through interaction with VEGF signaling pathways. For example, miR-140-5p has been shown to inhibit angiogenesis and invasion by directly targeting VEGF-A, leading to reduced expression of angiogenic markers such as CD31 and MMP-9. These findings indicate that certain miRNAs can directly interfere with the molecular machinery responsible for vascular development (76). Similarly, miR-204 has been implicated in the regulation of both angiogenesis and vasculogenic mimicry, particularly in breast cancer stem-like cells. Restoration of miR-204 expression has been associated with reduced formation of vascular-like structures and decreased levels of VEGF-A and β-catenin (77). This suggests that miRNAs may regulate not only classical angiogenesis but also alternative vascularization mechanisms within tumors.

In contrast, some miRNAs appear to promote angiogenesis and are associated with more aggressive tumor phenotypes. For instance, miR-155 has been shown to enhance angiogenesis by targeting the tumor suppressor VHL, leading to increased endothelial cell proliferation, migration, and network formation (78). Elevated miR-155 expression has also been associated with poor prognosis and advanced disease stage, suggesting its involvement in promoting a pro-angiogenic tumor microenvironment. Likewise, reduced expression of miR-206 has been linked to increased VEGF levels and enhanced angiogenesis and invasion in breast cancer, particularly in triple-negative subtypes (79). Restoration of miR-206 expression has been shown to suppress these processes, indicating that this miRNA may function as a negative regulator of angiogenesis. This observation aligns with findings from exercise-based studies, where increased miR-206 expression has been reported, suggesting a potential connection between systemic interventions and tumor-level molecular regulation (79). Hypoxia represents another critical factor influencing angiogenesis. Under low oxygen conditions, HIF-1α plays a central role in inducing VEGF expression and promoting vascular adaptation. In this context, miRNAs may act as modulators of hypoxia-responsive signaling. For example, miR-20b has been shown to regulate VEGF expression by targeting both HIF-1α and STAT3, thereby influencing transcriptional activation of angiogenic genes (80). This suggests that miRNAs may function as intermediaries between hypoxic stress and angiogenic signaling pathways. In addition, transcriptional regulators may influence angiogenesis through miRNA-mediated networks. The FOXO3a-miRNA axis provides an example of such regulation, where FOXO3a induces miR-29b-2 and miR-338, which in turn target VEGF-A and its co-receptor NRP1 (81).

This coordinated regulation has been associated with reduced angiogenesis, invasion, and metastasis, suggesting that upstream transcription factors may exert their effects through miRNA intermediates. Overall, the available evidence indicates that miRNAs play a multifaceted role in regulating angiogenesis, hypoxia response, and tumor microenvironment remodeling in breast cancer. These molecules appear to act at multiple levels, including direct targeting of VEGF signaling, modulation of hypoxia-responsive pathways, and integration of inflammatory signals. Exercise-related studies, particularly those by (70–73), suggest that systemic interventions may influence some of these processes, although the precise mechanisms remain to be fully elucidated. In conclusion, the regulation of angiogenesis and hypoxia-related pathways by miRNAs constitutes a complex, context-dependent facet of breast cancer progression. Although current findings suggest significant connections between exercise, miRNA expression, and tumor vascularization, further investigation is needed to clarify these interactions and their potential therapeutic applications. A comparative summary of both mechanistic and clinical evidence is provided in **Table 2**.

**Table 2:** Integrated summary of exercise-responsive microRNAs in breast cancer: Evidence from preclinical and clinical studies.

Exercise/Intervention type	miRNAs involved	Sample size and type	Key findings	Implications for treatment/detection	Reference
Interval exercise + Hormone therapy	miR-21, miR-206, let-7a	64 mice with breast tumor	Tumor size reduced, miR-21 down-regulated, miR-206 up-regulated	Anti-angiogenesis effect, potential therapeutic target for tumor growth regulation	(70)
Interval exercise + Tamoxifen	miR-21	48 mice (estrogen receptor-positive BC model)	Tumor size reduced, miR-21 expression down-regulated	Potential miR-21 as therapeutic marker in breast cancer treatment	(71)
Endurance exercise	miR-21, STAT3, Bcl2	20 mice	MiR-21 down-regulated, tumor volume decreased	Inhibition of miR-21/STAT3 pathway, suggesting benefit for tumor growth reduction	(72)
Aerobic exercise	miR-21, VEGF, HIF-1α	16 mice with breast cancer	Tumor size reduced, miR-21 down-regulated, apoptosis increased	Aerobic training reduced angiogenesis and promoted tumor suppression	(73)
High-intensity interval training	miR-21, miR-155, miR-221	15–26 women with breast cancer	MiR-21 increased, miR-155 and miR-221 decreased	HIIT with HT modifies miRNA expression and could help guide treatment choices	(137)
Weight loss + Exercise	miR-191-5p, miR-122-5p	121 BC survivors	MiR-191-5p significantly increased, BMI correlated with miRNA expression	Potential use of miRNAs as biomarkers for obesity and BC recurrence prediction	(138)
Lifestyle intervention (diet and exercise)	miR-10a-5p, miR-211-5p	84 women with metastatic BC	MiR-10a-5p and miR-211-5p upregulated in responders	Early indicators of treatment response in metastatic BC patients	(139)

STAT, signal transducer and activator of transcription; BMI, body mass index; BC, breast cancer; HIIT, high-intensity interval training; HT, hormone therapy; miR, microRNA; VEGF, vascular endothelial growth factor.

### 5.3 MicroRNA-orchestrated immune modulation, metabolic reprogramming, and epigenetic regulation in breast cancer

The progression of breast cancer is strongly influenced by coordinated changes in immune regulation, metabolic adaptation, and epigenetic remodeling within the tumor microenvironment. miRNAs have emerged as integrative regulators connecting these processes, acting at both tumor-intrinsic and tumor-extrinsic levels. Rather than functioning in isolation, these small non-coding RNAs appear to orchestrate networks that influence immune surveillance, cellular metabolism, and gene expression programs (82, 83). A growing body of evidence indicates that miRNAs contribute significantly to shaping the immune microenvironment in breast cancer. Elevated expression of miR-143 has been associated with a more favorable immune contexture, characterized by increased infiltration of anti-tumor immune cells and reduced presence of pro-tumor populations. This immune profile is accompanied by enrichment of Th1-related signaling and appears to correlate with improved survival outcomes, suggesting that miR-143 may be linked to a more immunologically active tumor phenotype (84). In parallel, circulating miRNAs such as miR-10b, miR-19a, miR-20a, miR-126, and miR-155 have been identified as potential indicators of immune-related disease progression and clinical outcome. Their differential expression patterns allow discrimination between early and metastatic stages and may reflect alterations in tumor–immune interactions (85). These observations suggest that miRNAs may function not only as regulators but also as measurable proxies of immune dynamics in breast cancer. Beyond their association with immune profiles, certain miRNAs appear to actively reprogram the tumor immune microenvironment. For example, miR-204-5p has been shown to suppress tumor proliferation and metastasis while simultaneously modulating immune-related pathways (86). Its overexpression influences cytokine production and alters the balance of immune cell populations within the tumor, indicating both cell-autonomous and non-cell-autonomous effects. Mechanistically, this miRNA targets key components of the PI3K/Akt pathway, linking intracellular signaling with immune modulation (86). In addition, tumor-derived miRNAs released into the interstitial fluid have been implicated in intercellular communication networks. Integrated analyses have revealed that specific secreted miRNAs are associated with immune gene clusters and lymphocyte infiltration, suggesting their role in coordinating immune responses within the tumor microenvironment. These findings support the concept that miRNAs can act as signaling molecules that mediate crosstalk between tumor cells and surrounding immune components (87).

The role of miRNAs in immune surveillance and immune escape further highlights their importance in cancer progression. Dysregulated miRNA expression can affect antigen presentation, interferon signaling, and immune checkpoint pathways, thereby influencing the balance between immune activation and suppression. Such alterations may enable tumor cells to evade immune detection while maintaining proliferative capacity. At the same time, restoration or modulation of specific miRNAs could potentially enhance anti-tumor immunity, although this remains to be validated in clinical settings (88). Recent findings also suggest that systemic factors, including physical activity, may interact with miRNA-mediated immune regulation. Exercise-induced release of extracellular vesicles containing miR-29a-3p has been shown to modify the tumor extracellular matrix by targeting collagen expression, thereby facilitating immune cell infiltration. This remodeling of the physical tumor environment appears to enhance T cell access and improve responsiveness to immunotherapy. Importantly, this mechanism links systemic physiological changes to local immune activation through a miRNA-dependent pathway, although further studies are needed to determine its generalizability across different tumor contexts (89). In addition to immune modulation, miRNAs play a crucial role in metabolic reprogramming of cancer cells, particularly through regulation of mitochondrial function. MiR-4485 has been identified as a mitochondria-associated miRNA capable of directly interacting with mitochondrial RNA and influencing its processing. Its expression affects ATP production, reactive oxygen species levels, and apoptotic signaling, ultimately leading to reduced tumorigenicity.

These findings suggest that miRNAs can directly regulate mitochondrial activity and energy metabolism, which are essential for tumor growth and survival (90). Similarly, miR-195 has been shown to modulate mitochondrial dynamics by targeting mitofusin-2, a key regulator of mitochondrial fusion. Increased expression of miR-195 leads to mitochondrial fragmentation and impaired respiratory function, which may contribute to apoptotic signaling and reduced cellular viability. This highlights the role of miRNAs in controlling mitochondrial structure and function, thereby influencing metabolic vulnerability in cancer cells (91). Additional evidence indicates that miRNA-mediated regulation of oxidative stress and mitochondrial dysfunction contributes to tumor suppression. Specific miRNAs, such as miR-4284, have been linked to regulation of mitochondrial proton leak and ROS production, leading to increased oxidative stress and DNA damage in cancer cells. These effects appear to reduce proliferative capacity and may represent a mechanism through which metabolic stress can be exploited for therapeutic purposes (92). Furthermore, miRNAs can regulate mitochondrial biogenesis and replication. For instance, miR-200a has been shown to target mitochondrial transcription factor A (TFAM), resulting in reduced mitochondrial DNA copy number and suppression of cell proliferation. This indicates that miRNAs can influence not only mitochondrial function but also mitochondrial content, thereby affecting overall cellular metabolism (93). Epigenetic regulation provides another layer of control over miRNA expression and function. The expression of certain tumor-suppressive miRNAs is regulated by histone modifications, which can lead to their silencing in cancer cells. For example, miR-125b-1 has been shown to be repressed through histone marks such as H3K27me3 and H3K9me3, and its reactivation can alter the expression of downstream targets involved in apoptosis (94). This suggests that epigenetic mechanisms can modulate miRNA availability and thereby influence tumor progression. More broadly, global epigenetic alterations have been associated with widespread changes in miRNA expression during breast carcinogenesis.

**Figure 1:**
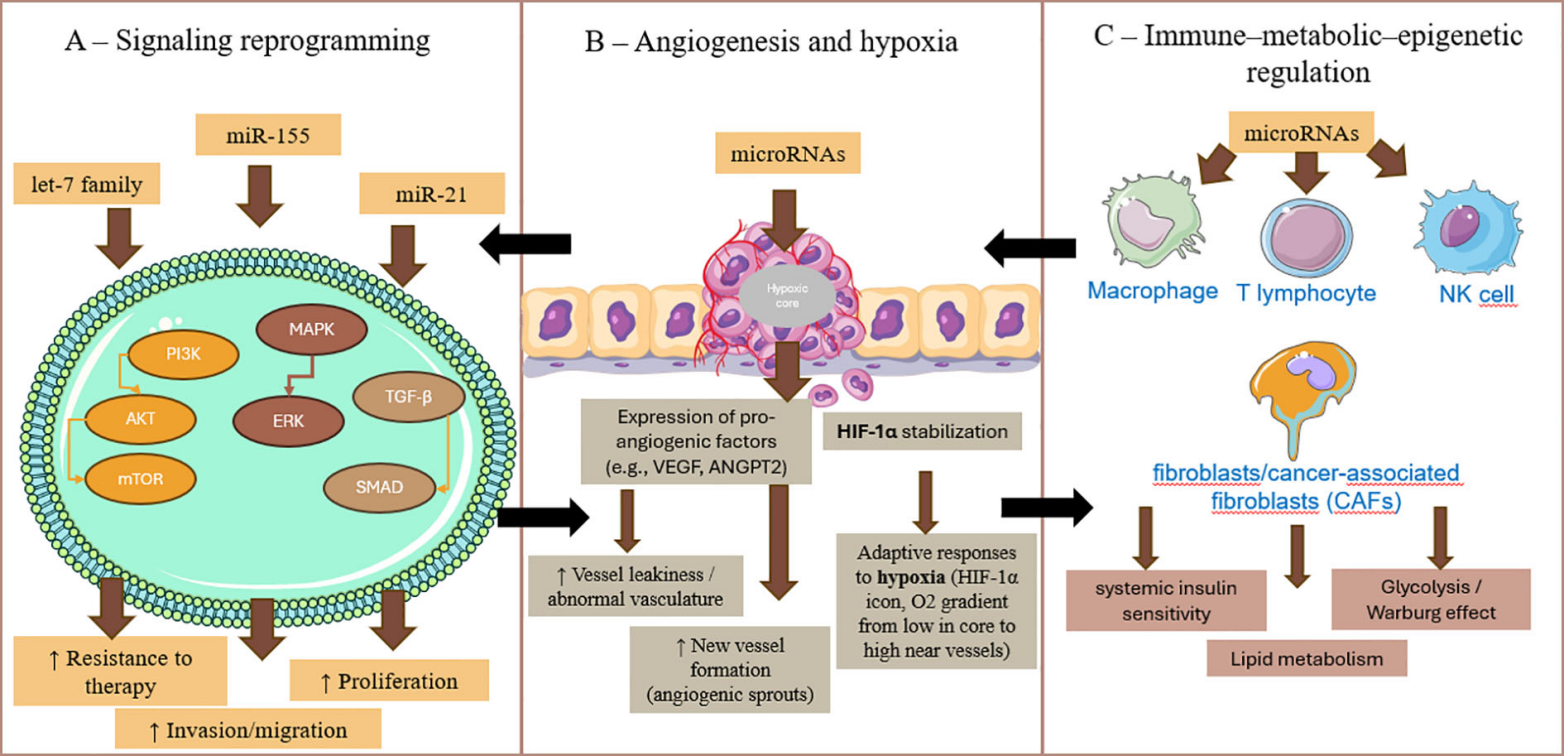
MicroRNA-driven multilayered regulation of breast cancer progression. **(A)** Signaling reprogramming. Dysregulated microRNAs (e.g., let-7 family, miR-155, and miR-21) modulate key oncogenic signaling pathways within breast cancer cells, including the PI3K/AKT/mTOR, MAPK/ERK, and TGF-β/SMAD cascades. Through post-transcriptional repression of pathway regulators and tumor-suppressor genes, these miRNAs reshape intracellular signaling networks, promoting increased proliferation, invasion and migration, as well as resistance to anticancer therapies. **(B)** Angiogenesis and hypoxia. MicroRNA-mediated regulation contributes to tumor vascular remodeling and adaptation to hypoxic stress. By influencing hypoxia-responsive pathways and stabilizing HIF-1α signaling, miRNAs enhance the expression of pro-angiogenic factors such as VEGF and ANGPT2. These changes promote angiogenic sprouting, abnormal and leaky vasculature, and adaptive cellular responses to oxygen gradients within the tumor microenvironment. **(C)** Immune–metabolic–epigenetic regulation. MicroRNAs also orchestrate interactions between tumor cells and the surrounding microenvironment. They regulate immune cell populations including macrophages, T lymphocytes, and NK cells, while modulating the activity of stromal components such as cancer-associated fibroblasts (CAFs). In parallel, miRNA networks influence metabolic reprogramming—affecting glycolysis (Warburg effect), lipid metabolism, and systemic insulin sensitivity—thereby linking immune modulation, metabolic adaptation, and epigenetic regulation to breast cancer progression.

These include DNA hypomethylation and alterations in histone modification patterns, which appear to occur early in tumor development and may contribute to both initiation and progression of disease. The interplay between epigenetic regulation and miRNA expression highlights a complex regulatory network that governs gene expression in cancer cells (95). Importantly, these molecular and epigenetic regulatory mechanisms do not operate in isolation but are influenced by systemic physiological conditions. In this context, systemic interventions such as exercise may interact with these regulatory networks through their effects on immune and metabolic pathways, thereby providing a functional link between molecular regulation and whole-body responses. Physical activity has been shown to enhance anti-tumor immune responses by promoting dendritic cell maturation, increasing Th1 polarization, and modulating cytokine production. More specifically, exercise appears to upregulate transcription factors such as T-bet while reducing GATA-3 and FoxP3 expression, indicating a shift away from immunosuppressive and Th2/Treg-associated profiles toward a more effective Th1-driven immune response (96). In parallel, exercise-induced changes in dendritic cells include increased expression of co-stimulatory and antigen-presenting molecules, which may improve T cell activation despite reductions in their absolute number within the tumor. These coordinated changes suggest that physical activity can reshape both innate and adaptive immune components, potentially alleviating immunosuppressive tumor microenvironments and enhancing anti-tumor immunity (96). In addition, exercise has been associated with increased cytotoxic activity of immune cells and improved response to chemotherapy, potentially through activation of apoptotic pathways and inflammatory signaling. Specifically, exercise training has been shown to enhance the infiltration and activation of natural killer (NK) cells and CD8^+^ T lymphocytes within tumor tissues, alongside increased expression of pro-inflammatory cytokines such as TNF-α and IFN-γ, which are known to support anti-tumor immunity (97). These immune changes appear to coincide with modulation of apoptotic regulators, including increased Bax and cleaved caspase-3 and reduced Bcl-2 expression, suggesting a shift toward pro-apoptotic signaling in tumor cells. Although these studies do not directly examine miRNA regulation, they suggest that systemic physiological changes can impact overlapping molecular pathways that are also influenced by miRNAs (97). In summary, miRNAs appear to function as central integrators of immune modulation, metabolic reprogramming, and epigenetic control in breast cancer. Through their ability to regulate multiple interconnected pathways, they contribute to the dynamic remodeling of the tumor microenvironment. While emerging evidence suggests that systemic factors such as exercise may interact with these regulatory networks, further studies are required to clarify their mechanistic relationships and therapeutic potential (**Figure 1****).**

## 6 Exercise-driven systemic non-coding RNA signaling: from circulating microRNAs to muscle-derived inter-organ communication

Exercise induces coordinated systemic adaptations that extend beyond local tissue responses, involving circulating and muscle-derived non-coding RNAs as key regulatory elements. These ncRNAs may participate in intercellular communication and metabolic regulation, suggesting a potential role in linking exercise stimuli to broader physiological and disease-relevant signaling pathways (98).

### 6.1 Dynamic regulation of circulating microRNAs in response to exercise: systemic signaling and adaptive responses

Circulating microRNAs (c-miRNAs) have emerged as stable, blood-based regulators that may reflect systemic physiological adaptations to exercise. Unlike intracellular miRNAs, these molecules can be detected in plasma and serum, suggesting a potential role in intercellular communication and endocrine-like signaling. Growing evidence indicates that exercise induces dynamic and context-dependent alterations in circulating miRNA profiles, which may be linked to vascular, metabolic, and inflammatory responses (99, 100). Acute resistance exercise has been shown to induce time-dependent changes in circulating miRNAs, indicating that their regulation is not immediate but evolves during the recovery phase. For example, specific miRNAs such as miR-149* (where the asterisk denotes the passenger strand derived from the same precursor miRNA duplex, also referred to as the miRNA*) increase several days after exercise, whereas others including miR-146a and miR-221 may decrease, suggesting differential regulatory patterns depending on their biological function (101). These delayed responses imply that circulating miRNAs may participate in post-exercise adaptation processes rather than acting solely as rapid stress indicators. In this context, miRNAs could contribute to longer-term remodeling mechanisms, potentially influencing gene expression networks involved in recovery and tissue adaptation (101). Beyond resistance exercise, endurance and exhaustive exercise protocols further highlight the dynamic nature of circulating miRNA responses. Distinct expression patterns have been identified, including miRNAs that respond consistently to acute exercise regardless of training status, those that lose responsiveness after chronic training, and those that are primarily modulated by long-term exercise exposure (102).

This classification suggests that circulating miRNAs may reflect both acute physiological stress and chronic adaptation, thereby serving as integrative markers of exercise exposure. Importantly, some miRNAs associated with angiogenesis, inflammation, and hypoxia pathways such as miR-20a, miR-21, and miR-146a, demonstrate context-specific responsiveness, indicating that exercise may selectively engage different biological pathways depending on training status and physiological demand (102).

The role of exercise intensity and duration in shaping circulating miRNA responses has also been investigated, revealing dose-dependent regulatory patterns. Certain miRNAs appear to increase only beyond specific thresholds of exercise intensity or duration, while others respond in a graded manner. For instance, muscle-enriched miRNAs such as miR-1 and miR-133a show sensitivity to exercise load, with changes in circulating levels potentially reflecting their release from skeletal muscle and corresponding intracellular depletion (103). This phenomenon suggests a coordinated redistribution of miRNAs between tissues and circulation, which may facilitate transcriptional reprogramming in response to exercise. Such dose-dependent behavior further supports the concept that circulating miRNAs are not merely byproducts of cellular stress but may actively participate in regulating adaptive responses (103). In addition to intensity and duration, different exercise modalities can differentially influence circulating miRNA profiles, particularly those associated with vascular biology. Variations in exercise protocols including high-volume, high-intensity, and sprint-based training, have been shown to produce distinct changes in endothelial-derived miRNAs such as miR-21 and miR-126 (104). These miRNAs are known to be involved in endothelial function and vascular remodeling, and their increased presence in circulation following exercise may indicate enhanced endothelial activation or turnover.

Furthermore, the packaging of these miRNAs into endothelial microparticles and their subsequent transfer to target cells suggest a mechanism for intercellular communication that extends beyond the site of origin. Such findings reinforce the idea that circulating miRNAs may act as mediators of systemic vascular adaptation to exercise (104). Even low-intensity exercise appears capable of modulating circulating miRNA profiles, highlighting the sensitivity of these molecules to relatively modest physiological stimuli. In individuals without prior exercise habits, short-term resistance training has been associated with upregulation of specific miRNAs such as miR-630 and miR-5703, alongside concurrent changes in cytokine and myokine expression (105). Pathway analysis of these miRNAs suggests potential involvement in signaling cascades such as TGF-β and Wnt pathways, which are known to regulate cellular proliferation and differentiation (105). The simultaneous alteration of miRNAs and inflammatory mediators further indicates that circulating miRNAs may be integrated within broader systemic signaling networks, linking metabolic and immune responses to exercise. Collectively, these findings suggest that circulating miRNAs are dynamically regulated by exercise in a manner that depends on intensity, duration, modality, and training status. Rather than representing passive biomarkers, c-miRNAs may contribute to systemic adaptation by facilitating communication between tissues and modulating key biological pathways. Although their direct role in disease contexts requires further investigation, the observed responsiveness of circulating miRNAs to exercise provides a potential mechanistic basis for understanding how systemic physiological changes could influence broader regulatory networks.

### 6.2 Muscle-derived microRNAs and extracellular vesicle–mediated inter-organ communication in exercise adaptation

Skeletal muscle is increasingly recognized not only as a contractile tissue but also as an active secretory organ capable of releasing regulatory molecules into the circulation. Among these, muscle-derived non-coding RNAs have been proposed to contribute to systemic communication during exercise. These molecules may coordinate local muscle adaptation with broader physiological responses, potentially linking metabolic, inflammatory, and inter-organ signaling pathways (106, 107). Exercise-induced modulation of myomiRs (muscle-specific microRNAs predominantly expressed in skeletal muscle and involved in the regulation of muscle development, homeostasis, and adaptation) appears to play a central role in maintaining muscle homeostasis under both physiological and pathological conditions. In cancer-associated contexts, alterations in muscle-specific miRNAs such as miR-486 and miR-206 have been observed in both skeletal muscle and circulation, suggesting coordinated regulation across tissues (108). Notably, differential expression patterns of these myomiRs between cachectic and non-cachectic states imply that their regulation may depend on disease context as well as exercise intervention. The observation that miR-206 levels change concurrently in muscle, serum, and tumor tissue further supports the possibility of a communication axis linking muscle and distant organs. Although exercise training may not fully reverse all molecular alterations, its ability to preserve muscle function alongside modulation of specific myomiRs suggests a partial protective role that may be mediated through these regulatory RNAs (108). Beyond circulating myomiRs, intracellular regulation of miRNAs within skeletal muscle contributes to metabolic adaptation to exercise. For instance, miR-761 has been identified as a negative regulator of mitochondrial biogenesis through its targeting of PGC-1α, a key transcriptional coactivator involved in oxidative metabolism (109). Exercise-induced downregulation of miR-761 appears to relieve this inhibition, thereby promoting mitochondrial function and enhancing energy metabolism in skeletal muscle. This regulatory mechanism highlights how miRNAs may act as molecular switches that fine-tune intracellular signaling pathways, including p38 MAPK-dependent transcriptional programs, in response to exercise stimuli (109). Such adaptations may not only support muscle performance but also influence systemic metabolic homeostasis.

Extracellular vesicles (EVs) have emerged as a critical mechanism for the transport of muscle-derived miRNAs into the circulation. Muscle tissue has been shown to release EVs enriched with specific miRNAs, including miR-206, which can be detected in the bloodstream following exercise (110). These vesicles, characterized by markers such as alpha-sarcoglycan and CD81, may represent a specialized subpopulation originating from skeletal muscle. The enrichment of muscle-specific miRNAs within these EVs and their association with aerobic fitness suggest that EV-mediated transport could facilitate targeted delivery of regulatory signals to distant tissues. In this context, EVs may function as carriers that protect miRNAs from degradation while enabling their participation in intercellular communication (110). However, the relationship between intracellular, circulating, and exosomal miRNAs appears to be complex and not always directly correlated. Distinct expression patterns have been observed across muscle tissue, plasma, and exosome fractions following exercise, with some miRNAs showing coordinated changes while others exhibit compartment-specific regulation (111). Notably, exosomal miRNAs tend to show a consistent increase after exercise, even when corresponding changes in muscle or plasma are variable. This discrepancy suggests that the packaging and release of miRNAs into EVs may be a selective and regulated process rather than a passive reflection of intracellular abundance. Such selective enrichment further supports the hypothesis that EV-associated miRNAs may have specific functional roles in systemic signaling (111). In parallel with ncRNA-mediated communication, cytokine signaling represents an additional layer of muscle-derived systemic regulation that may interact with miRNA pathways. Exercise stimulates the release of multiple cytokines, including IL-6, IL-8, IL-10, and TNF-α, which are involved in metabolic and inflammatory processes (112).

**Figure 2:**
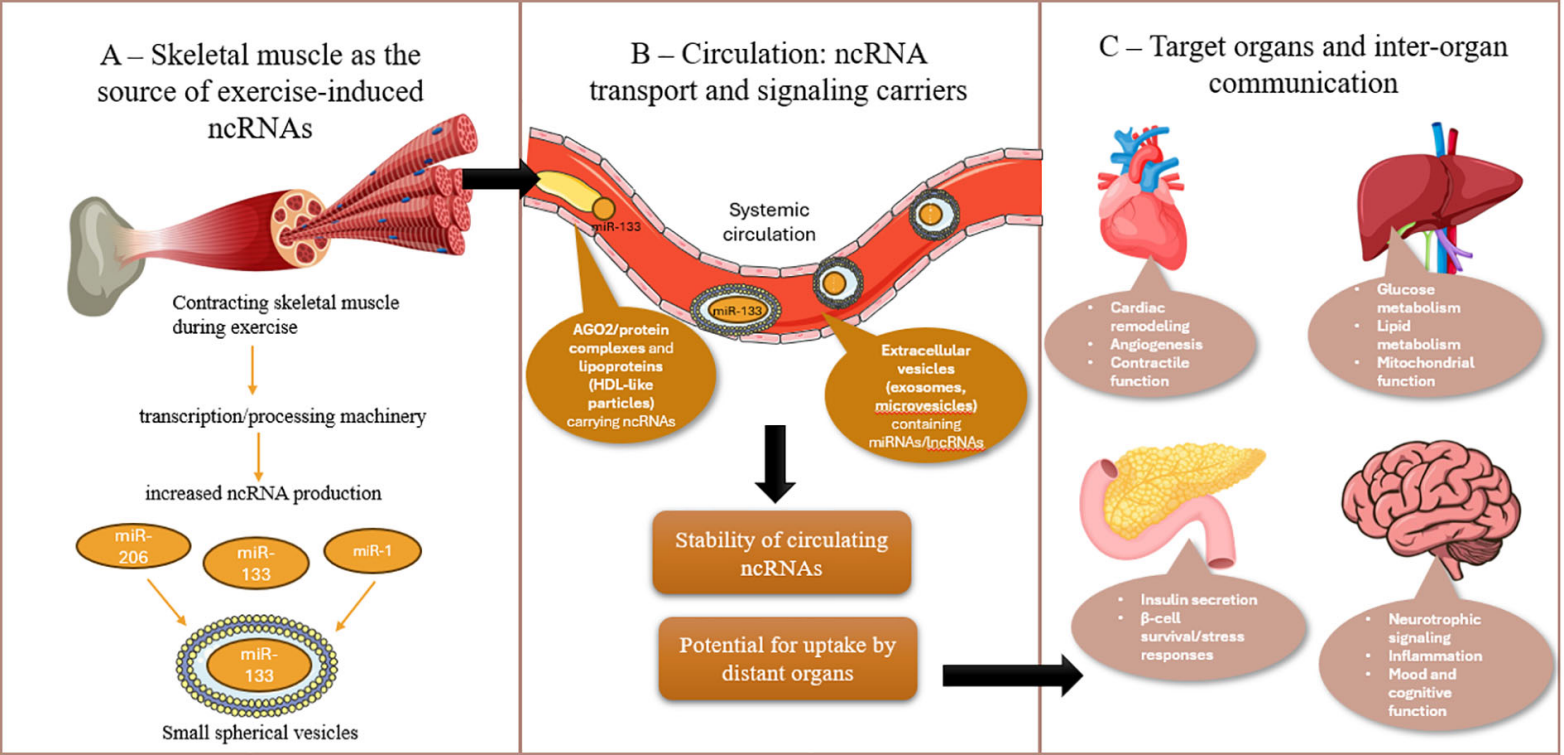
Exercise−driven systemic non−coding RNA signaling and muscle−derived inter−organ communication. **(A)** Skeletal muscle-derived exercise-responsive ncRNAs. During physical activity, contracting skeletal muscle activates transcriptional and post−transcriptional regulatory mechanisms that increase the production of muscle−enriched non−coding RNAs (ncRNAs), including microRNAs such as miR−1, miR−133, and miR−206. These ncRNAs are selectively packaged into small extracellular vesicles or associated with protein complexes, enabling their release from muscle fibers into the extracellular environment. **(B)** Circulating ncRNA transport and signaling. Exercise−induced ncRNAs enter the systemic circulation where they are stabilized by carrier systems such as extracellular vesicles (exosomes and microvesicles), Argonaute−2 (AGO2) protein complexes, and lipoprotein particles. These carriers protect ncRNAs from degradation and facilitate their transport through the bloodstream, allowing them to function as endocrine−like signaling molecules capable of reaching distant tissues. **(C)** Target organs and inter−organ communication. Circulating exercise−responsive ncRNAs can be taken up by multiple organs, including the heart, liver, pancreas, and brain. In these tissues, ncRNAs regulate diverse physiological processes such as cardiac remodeling, angiogenesis and contractile function, metabolic regulation in the liver, insulin secretion and β−cell stress responses in the pancreas, and neurotrophic signaling, inflammation, and cognitive function in the brain. Through these mechanisms, muscle−derived ncRNAs contribute to systemic metabolic adaptation and the broad health benefits associated with regular physical activity.

The production and release of these cytokines are tightly regulated by intracellular signaling networks, including those influenced by miRNAs and RNA-binding proteins. Although not all circulating cytokines originate from skeletal muscle, the coordinated regulation of cytokines and miRNAs suggests the existence of integrated signaling networks that link muscle activity to systemic physiological responses (112). These interactions may contribute to the modulation of immune and metabolic pathways in response to exercise. Taken together, current evidence indicates that muscle-derived ncRNAs and EV-mediated transport mechanisms may play an important role in coordinating local and systemic responses to exercise. Through the combined action of myomiRs, intracellular regulatory pathways, and vesicle-mediated communication, skeletal muscle may influence distant tissues and contribute to whole-body adaptation. While the precise functional implications of these pathways require further clarification, these findings provide a mechanistic framework for understanding how exercise-induced signals could extend beyond muscle to impact broader biological systems (**Figure 2****).**

## 7 EV–mediated microRNA signaling in breast cancer: functional roles and clinical implications

EV-associated microRNAs have emerged as critical mediators of intercellular communication in breast cancer, influencing both tumor progression and systemic responses. By enabling the transfer of regulatory signals between tumor and host cells, EV-miRNAs contribute to key processes such as therapy resistance, metabolic reprogramming, and disease dissemination. In parallel, their stability and accessibility in body fluids position them as promising candidates for non-invasive biomarkers in diagnosis, prognosis, and treatment monitoring (113, 114).

### 7.1 EV-mediated microRNA signaling in tumor progression and therapeutic response

EVs have emerged as key mediators of intercellular communication in cancer, enabling the transfer of regulatory molecules such as microRNAs between tumor cells and distant tissues. Through selective packaging and release, EV-associated miRNAs may influence diverse biological processes, including metabolic reprogramming, therapy response, and tumor–host interactions. Increasing evidence suggests that these vesicle-mediated signals are not merely byproducts of tumor activity but may actively contribute to disease progression (115, 116). One of the most compelling examples of EV-mediated tumor–host interaction involves cancer-derived miRNAs that modulate distant organ function. Tumor-secreted EVs carrying miR-122 have been shown to disrupt skeletal muscle homeostasis by targeting O-GlcNAc transferase (OGT), thereby reducing protein O-GlcNAcylation levels in muscle tissue (117). This reduction alters the balance between phosphorylation and ubiquitination of key structural proteins such as ryanodine receptor 1, ultimately leading to increased proteasomal degradation and activation of calcium-dependent proteolytic pathways. These molecular changes contribute to muscle wasting and impaired contractility, highlighting how tumor-derived EV miRNAs can induce systemic metabolic alterations beyond the primary tumor site. Such findings support the concept that EV-mediated signaling participates in the development of cancer-associated cachexia through long-distance regulatory mechanisms (117). In addition to systemic metabolic effects, EV-associated miRNAs play a critical role in modulating therapeutic resistance within the tumor microenvironment.

In breast cancer, miR-222 has been identified as a key regulator of anthracycline resistance through its targeting of the PTEN/FN1 signaling axis (118). Elevated levels of miR-222 in resistant cells are associated with altered tumor-infiltrating immune profiles and enhanced survival pathways. Notably, engineered EVs designed to deliver miR-222 inhibitors have been shown to restore PTEN expression and suppress tumor growth in resistant models, suggesting a potential therapeutic strategy based on EV-mediated RNA delivery (118). These findings indicate that EVs can function both as carriers of resistance-promoting signals and as vehicles for targeted therapeutic intervention, depending on their molecular cargo. Lifestyle-related interventions may also influence EV-mediated miRNA signaling, providing an additional layer of regulation in cancer-related pathways. Dietary modulation, such as adherence to a Mediterranean diet, has been associated with significant changes in EV-associated miRNA profiles in breast cancer survivors (119). These alterations include miRNAs linked to metabolic and oncogenic pathways, such as those involved in glucose metabolism, insulin signaling, and tumor progression. Although the direct functional consequences of these changes remain to be fully elucidated, the observed shifts in EV miRNA signatures suggest that systemic factors may modulate EV-mediated communication networks. This raises the possibility that non-pharmacological interventions could indirectly influence tumor-related signaling through regulation of circulating vesicle-associated miRNAs (119). Taken together, these studies suggest that EV-mediated miRNAs contribute to multiple dimensions of cancer biology, including tumor–host metabolic interactions, therapeutic resistance, and systemic regulation of signaling pathways. Through targeted delivery of specific miRNAs, EVs may facilitate communication between tumors and distant tissues, thereby shaping both local and systemic disease processes. Despite growing evidence on EV-mediated miRNA signaling in breast cancer and exercise-induced modulation of circulating ncRNAs, direct mechanistic studies integrating these axes remain limited.

### 7.2 Liquid biopsy potential of exosomal microRNAs in breast cancer: from early detection to therapy response

Exosomal microRNAs have attracted considerable attention as minimally invasive biomarkers due to their stability in circulation and their ability to reflect the molecular characteristics of tumor cells. Unlike free-circulating miRNAs, exosome-encapsulated miRNAs are selectively packaged and protected from degradation, which may enhance their reliability for clinical applications in breast cancer diagnosis and disease monitoring (120). Initial evidence supporting the diagnostic potential of exosomal miRNAs has demonstrated that specific miRNA species are selectively enriched in tumor-derived vesicles and can be detected in patient plasma. For instance, miR-1246 and miR-21 have been identified at significantly elevated levels in plasma exosomes of breast cancer patients compared to healthy individuals, suggesting their potential utility as disease indicators (121). Importantly, the combined assessment of multiple exosomal miRNAs appears to improve diagnostic accuracy compared to single markers, highlighting the value of multi-miRNA signatures in capturing tumor heterogeneity. These findings support the concept that exosomal miRNAs may serve as tumor-specific molecular fingerprints accessible through liquid biopsy approaches (121). Beyond diagnosis, exosomal miRNAs have also been associated with disease progression and clinical outcomes. Systematic analyses have shown that various exosomal components, including miRNAs and proteins, are correlated with key prognostic parameters such as overall survival, disease-free survival, metastasis, and recurrence (122).

Specific miRNAs, including miR-1246 and miR-21, have been linked to more aggressive disease phenotypes, while others are associated with chemotherapy resistance and treatment failure. These observations suggest that exosomal miRNAs may not only indicate the presence of disease but also provide insight into tumor behavior and patient prognosis (122). The predictive value of exosomal miRNAs has been further explored in the context of treatment response, particularly in patients undergoing neoadjuvant chemotherapy. Distinct expression patterns of miRNAs such as miR-21, miR-105, and miR-222 have been associated with tumor subtype, proliferation markers, and the presence of circulating tumor cells (123). Changes in exosomal miRNA levels during treatment have also been shown to correlate with tumor size and proliferative activity, indicating their potential utility in monitoring therapeutic response (123). Similarly, specific miRNAs, including miR-30b, miR-328, and miR-423, have been identified as potential predictors of pathological complete response, while others may indicate poor response to therapy. Although these findings require validation in larger cohorts, they suggest that exosomal miRNAs could contribute to personalized treatment strategies by enabling early prediction of therapeutic outcomes (124). In addition to treatment response, exosomal miRNAs have demonstrated potential as biomarkers for disease recurrence. Comparative analyses between patients with and without recurrence have identified distinct miRNA expression profiles, including the upregulation of miR-340-5p and downregulation of miR-195-5p and miR-93-5p in recurrent cases (125). Interestingly, discrepancies between miRNA expression in tumor tissues and circulating exosomes have been observed, suggesting that cancer cells may selectively release specific miRNAs into the extracellular environment. This selective export mechanism may reflect an adaptive strategy by tumor cells to modulate their microenvironment or systemic signaling pathways, further supporting the functional relevance of exosomal miRNAs (125).

Exosomal miRNAs may also enable early detection of disease progression and invasive transformation. For example, miR-223-3p has been identified as a potential biomarker for distinguishing invasive ductal carcinoma from non-invasive lesions, with elevated levels observed in patients with more advanced disease (126). Its association with clinicopathological features such as tumor stage, lymphatic invasion, and nuclear grade further underscores its potential as an indicator of tumor aggressiveness. These findings suggest that exosomal miRNAs may provide clinically relevant information even at early stages of disease development (126). In parallel, certain exosomal miRNAs have demonstrated strong diagnostic performance in distinguishing breast cancer patients from healthy individuals. Reduced levels of serum exosomal miR-200c, for instance, have been consistently observed in breast cancer patients and shown to possess significant discriminative power in independent cohorts (127). The combination of exosomal miRNAs with conventional biomarkers has also been proposed to enhance diagnostic accuracy, indicating a potential integrative approach for clinical application. Such strategies may improve early detection and facilitate more accurate disease classification (127). Taken together, current evidence suggests that exosomal microRNAs represent a promising class of biomarkers for breast cancer diagnosis, prognosis, treatment response, and recurrence monitoring. Their selective packaging, stability, and association with tumor-derived processes provide unique advantages for non-invasive assessment. However, variability in detection methods, cohort size, and standardization of analytical approaches may influence their clinical applicability. Further large-scale and longitudinal studies are required to validate these findings and establish robust frameworks for their integration into routine clinical practice.

## 8 Bidirectional muscle–tumor crosstalk mediated by systemic ncRNAs: from exercise-induced tumor suppression to cancer cachexia

Skeletal muscle and tumors engage in dynamic, bidirectional communication that extends beyond local tissue interactions and involves systemic molecular signaling. Non-coding RNAs, particularly those transported through circulation and extracellular vesicles, have emerged as key mediators of this crosstalk. While exercise reprograms muscle to generate signals with potential anti-tumor effects, tumors can reciprocally induce catabolic pathways in muscle, contributing to cachexia. This section integrates these opposing yet interconnected processes to highlight a unified framework of ncRNA-driven inter-organ communication in cancer.

### 8.1 Exercise-reprogrammed muscle signaling: systemic ncRNA networks in tumor suppression

Skeletal muscle is increasingly recognized as an active regulator of tumor biology rather than a passive recipient of cancer-induced alterations. Through exercise-driven systemic signaling, muscle-derived ncRNAs along with circulating exerkines and secretome factors, contribute to an integrated anti-tumor network. Collectively, these signals may reprogram the systemic milieu, thereby modulating tumor growth, survival, and therapeutic responsiveness (128). One of the most compelling mechanistic links in this axis is provided by miR-486, a muscle-enriched microRNA with established roles in maintaining muscle integrity. Experimental overexpression of miR-486 in skeletal muscle has been shown to mitigate tumor-induced functional decline, including impairments in contractile force and motor performance. Importantly, these muscle-specific molecular alterations were accompanied by partial suppression of tumor growth and improved survival outcomes, suggesting a systemic effect extending beyond local muscle preservation. Mechanistically, miR-486 was associated with restoration of key signaling pathways such as PI3K/AKT and modulation of inflammatory mediators, including IL-6, indicating that muscle-derived miRNAs may influence tumor progression indirectly through systemic inflammatory and metabolic regulation (129). Complementing this molecular perspective, functional studies using exercise-conditioned human serum and muscle-derived secretome further support the concept of muscle-to-tumor communication. Circulating factors obtained following structured exercise interventions have been shown to reduce cancer cell viability and induce apoptotic signaling in breast cancer models. These effects were accompanied by increased cytotoxicity, enhanced DNA damage, and downregulation of pro-survival mediators such as IL-8 and VEGF. Notably, similar outcomes were observed when tumor cells were exposed to secretome derived from mechanically stimulated myotubes, suggesting that muscle contraction itself can generate bioactive signals with anti-tumor properties. These findings indicate that exercise-induced systemic factors may function as mediators of intercellular signaling capable of directly influencing tumor cell fate (130). At a broader systems level, transcriptomic analyses provide further insight into how exercise reprograms muscle-derived signaling with potential anti-cancer implications. High-intensity aerobic exercise has been shown to induce widespread changes in skeletal muscle gene expression, including the upregulation of genes with tumor-suppressive functions.

Functional validation of selected exercise-responsive genes demonstrated their capacity to reduce cancer cell proliferation, suggesting that muscle-derived molecular outputs can exert measurable effects on tumor viability. These observations support the notion that exercise triggers a coordinated remodeling of the muscle transcriptome, generating a repertoire of circulating factors that may contribute to systemic anti-tumor adaptation (131). Taken together, these findings converge on a conceptual framework in which exercise acts as a physiological stimulus that reprograms skeletal muscle into a source of systemic regulatory signals, including ncRNAs and secreted factors, capable of modulating tumor behavior. While the precise contribution of individual components remains to be fully elucidated, the integration of myomiRs, exerkines, and gene expression changes suggests a multi-layered communication network. This perspective moves beyond a purely descriptive view and highlights the potential of exercise-driven muscle signaling as an active participant in tumor regulation, thereby opening new avenues for mechanistic understanding and therapeutic exploitation.

### 8.2 Tumor-to-muscle communication: exosomal ncRNAs and inflammatory signaling in cancer cachexia

In contrast to exercise-driven protective signaling, accumulating evidence indicates that tumors actively reprogram skeletal muscle through systemic molecular communication, contributing to the development of cancer cachexia. This process is not merely a consequence of nutrient imbalance but rather reflects a coordinated signaling network in which tumor-derived factors, including exosomal ncRNAs and inflammatory mediators, disrupt muscle homeostasis and promote catabolic remodeling (132). One of the key mechanistic insights into this axis involves the suppression of muscle-enriched microRNAs by tumor-derived signals. Circulating levels of miR-486, a critical regulator of muscle integrity, have been shown to decline significantly in cancer conditions, both in patients and in preclinical tumor models. This reduction is accompanied by upregulation of its target genes, such as PTEN and FOXO1, and attenuation of PI3K/AKT signaling, ultimately impairing muscle differentiation and survival pathways. Notably, exposure of muscle cells to tumor-conditioned media was sufficient to reproduce these molecular alterations, implicating soluble tumor-derived factors in this regulatory process. Cytokine profiling further identified inflammatory mediators, including TNF-α, as upstream drivers of miRNA dysregulation, highlighting the integration of inflammatory and ncRNA signaling in tumor-induced muscle dysfunction (133). Beyond individual miRNAs, exosome-mediated transfer of regulatory molecules has emerged as a central mechanism underlying tumor–muscle communication. Exosomes serve as stable carriers of miRNAs and myomiRs, protecting them from degradation and enabling targeted delivery to distant tissues. Within the context of cachexia, tumor-derived exosomal miRNAs have been implicated in the activation of proteolytic pathways, modulation of inflammatory signaling, and disruption of protein synthesis in skeletal muscle. These vesicle-mediated interactions facilitate a highly efficient form of intercellular communication, allowing tumors to orchestrate systemic metabolic alterations that favor disease progression. Importantly, several exosomal miRNAs have been associated with pathways regulating muscle atrophy, inflammation, and tumor aggressiveness, suggesting that they operate at the intersection of tumor biology and host tissue remodeling (134). Complementary evidence from human muscle transcriptomic profiling further supports the role of ncRNAs in cachexia-associated muscle alterations. Comparative analyses between cachectic and non-cachectic cancer patients have identified distinct sets of differentially expressed miRNAs linked to pathways involved in myogenesis, inflammation, and metabolic regulation.

These miRNAs are predicted to target a wide range of genes associated with muscle structure and function, providing mechanistic insight into the molecular basis of muscle wasting. Moreover, the identified miRNA signatures exhibit potential prognostic and predictive value, indicating that muscle-derived ncRNA alterations may reflect disease severity and progression (135). At a broader level, tumor-derived exosomal miRNAs have been proposed as key drivers of cancer-associated sarcopenia through their ability to induce systemic catabolic signaling. These miRNAs can promote inflammatory cascades, enhance proteolysis, and interfere with mitochondrial function, collectively contributing to progressive muscle loss. The integration of exosomal signaling with intracellular regulatory networks suggests a multi-layered mechanism in which tumors exploit circulating vesicles to reprogram distant tissues. This perspective reinforces the concept that muscle wasting in cancer is not solely a local phenomenon but rather the outcome of dynamic and bidirectional communication between tumor and host (136). Taken together, these findings delineate a mechanistic framework in which tumor-derived exosomal ncRNAs, in conjunction with inflammatory mediators, drive skeletal muscle dysfunction and cachexia. Importantly, this tumor-to-muscle signaling axis stands in direct contrast to exercise-induced protective pathways, underscoring a dynamic balance between catabolic and anabolic systemic cues. Understanding this interplay not only provides deeper insight into cancer-associated muscle wasting but also highlights potential therapeutic targets aimed at disrupting maladaptive inter-organ communication.

## 9 Circulating microRNAs in breast cancer: from treatment response to early detection and prognostic stratification

The involvement of circulating miRNAs in breast cancer has garnered significant attention due to their potential to reflect dynamic biological processes underlying tumor progression and treatment response. miRNAs, as non-invasive biomarkers, offer insights into both the molecular mechanisms of disease and the impact of lifestyle interventions on tumor behavior. Exploring their role in early detection, therapeutic modulation, and prognostic stratification provides a comprehensive understanding of their clinical utility across different stages of breast cancer.

### 9.1 Exercise- and weight-loss-modulated circulating microRNAs as indicators of treatment response in breast cancer

Accumulating clinical data suggest that circulating miRNAs could serve as responsive molecular indicators, reflecting how breast cancer patients adapt to lifestyle interventions alongside conventional therapies. In this context, structured exercise and weight-management strategies are increasingly recognized not only as supportive measures to enhance physical function and metabolic status but also as modulators of systemic signaling pathways associated with tumor behavior and therapeutic responsiveness. While current evidence remains somewhat limited and heterogeneous, emerging findings imply that changes in circulating miRNA profiles may capture biologically significant alterations across various stages of the disease. When interpreted alongside mechanistic data, these findings further underscore the translational relevance of exercise-responsive miRNAs in both experimental and clinical settings (**Table 2**). In this context, Alizadeh et al. (137) investigated the impact of HIIT on circulating miRNA expression in women undergoing hormone therapy for hormone receptor-positive breast cancer. Their results demonstrated that, compared with healthy individuals, patients exhibited a dysregulated miRNA signature characterized by elevated levels of oncogenic miRNAs—including miR-21, miR-155, miR-221, miR-27a, and miR-10b—and reduced expression of tumor-suppressive miRNAs such as miR-206, miR-145, miR-143, miR-9, and let-7a. While hormone therapy alone partially mitigated this imbalance, the integration of a 12-week HIIT program led to a more pronounced normalization, further suppressing oncomiRs and enhancing tumor-suppressive miRNAs. These findings imply that exercise may reinforce treatment-induced molecular shifts and highlight the potential of circulating miRNAs as dynamic indicators of therapeutic adaptation (137). From a complementary perspective, Adams et al. (138) explored the interplay between adiposity, weight reduction, and circulating miRNA expression in breast cancer survivors across two independent clinical cohorts. Their analyses revealed significant associations between baseline body mass index (BMI) and specific circulating miRNAs, including a negative correlation with miR-191-5p and a positive correlation with miR-122-5p, suggesting that metabolic status may influence systemic miRNA patterns. Notably, during a structured weight-loss intervention, miR-191-5p levels exhibited an upward trend, and integrative pathway analysis linked intervention-responsive miRNAs to key oncogenic and endocrine-related pathways, including estrogen-mediated cell cycle regulation and broader cancer-associated signaling networks. These observations support the notion that exercise and weight reduction may induce measurable molecular changes that extend beyond general physiological benefits, potentially reflecting modifications in pathways implicated in tumor progression and recurrence risk. However, given the relatively modest effect sizes reported, cautious interpretation is warranted pending further validation (138).

Additional support for the clinical relevance of circulating miRNAs is provided by studies in advanced disease settings. Olson et al. (139) evaluated circulating miRNA profiles in women with metastatic breast cancer undergoing lifestyle interventions focused on dietary improvement and increased physical activity. Their findings indicated that miR-10a-5p and miR-211-5p were downregulated in nonresponders but upregulated in responders, whereas miR-205-5p increased in both groups, with a more pronounced elevation among responders. Furthermore, an inverse association between miR-10a-5p and the inflammatory marker IL-6 was observed, suggesting a potential link between miRNA modulation and systemic inflammatory dynamics. These results raise the possibility that specific circulating miRNAs may serve as early indicators of responsiveness to lifestyle interventions, particularly in metastatic contexts where timely evaluation of intervention efficacy is clinically valuable (139). Collectively, these studies point toward an emerging role for circulating miRNAs as sensitive, non-invasive biomarkers capable of capturing exercise- and weight-loss-induced molecular adaptations in breast cancer. Across different clinical scenarios including adjuvant therapy, survivorship, and metastatic disease, changes in miRNA profiles appear to parallel shifts in oncogenic signaling, metabolic regulation, and inflammatory status. While further large-scale and longitudinal investigations are necessary to establish their clinical utility, current evidence suggests that circulating miRNAs may contribute to more refined monitoring of treatment response and personalized lifestyle-based interventions in breast cancer management.

### 9.2 Circulating microRNAs in breast cancer: from early detection and risk prediction to prognostic stratification

Accumulating clinical evidence indicates that circulating miRNAs hold considerable promise as minimally invasive biomarkers across multiple stages of breast cancer management. Their remarkable stability in biological fluids, coupled with their regulatory involvement in tumorigenesis, has positioned them as attractive candidates for early detection, risk prediction, and prognostic evaluation. Importantly, recent clinical studies suggest that distinct miRNA signatures may capture different dimensions of disease biology, ranging from preclinical risk states to aggressive tumor phenotypes and relapse dynamics. In the context of early detection, several investigations have focused on identifying circulating miRNA signatures capable of distinguishing breast cancer patients from healthy individuals. A comprehensive study by Yu et al. (140) employed a tissue-informed discovery approach followed by serum validation, resulting in the identification of a three-miRNA panel (miR-21-3p, miR-21-5p, and miR-99a-5p) with high diagnostic performance. The integration of tissue-derived data with circulating biomarkers strengthened the biological relevance of this signature and demonstrated its potential utility as a non-invasive screening tool. Complementing this multi-marker strategy, Sun et al. (141) evaluated serum miR-155 and reported significantly elevated levels in breast cancer patients compared with controls. Notably, miR-155 also exhibited a decline following surgical and chemotherapeutic interventions, suggesting that certain circulating miRNAs may serve dual roles in both diagnosis and therapeutic monitoring. Beyond blood-based biomarkers, alternative biological fluids have also been explored to enhance non-invasive detection strategies. Hirschfeld et al. (142) demonstrated that urinary exosomal miRNAs could effectively discriminate breast cancer patients from healthy individuals using a defined four-miRNA panel (miR-424, miR-423, miR-660, and let-7i).

The exceptionally high sensitivity and specificity reported in this study highlight the potential of urine-based assays as complementary or even alternative screening modalities. Similarly, Bakr et al. (143) identified miR-373 as an upregulated circulating oncomiR associated with breast cancer presence and clinicopathological characteristics. Importantly, its correlation with key oncogenic targets such as VEGF and cyclin D1 provides mechanistic support for its diagnostic relevance, linking biomarker detection to angiogenic and proliferative signaling pathways. In addition to detecting established disease, circulating miRNAs may also provide insight into long-term cancer risk before clinical manifestation. A prospective cohort analysis by Muti et al. (144) revealed that altered leukocyte miRNA profiles could be detected years prior to breast cancer diagnosis. Among these, miR-513a-5p emerged as a significant risk-associated miRNA, with its upregulation correlating with increased disease risk and hormonal factors, including progesterone and testosterone levels. This finding is particularly important, as it suggests that circulating miRNAs may not only serve as passive indicators of disease presence but also actively reflect endocrine-driven carcinogenic processes. Such evidence supports the potential integration of miRNA profiling into risk stratification frameworks for apparently healthy populations (144). While early detection and risk prediction are critical, the clinical utility of circulating miRNAs extends further into prognostic assessment and disease monitoring. To summarize the key circulating miRNAs associated with early detection, risk prediction, and prognostic stratification in breast cancer, **Table 3** provides a detailed overview of these non-invasive biomarkers. A large-scale study by Gahlawat et al. (145) demonstrated that total circulating cell-free miRNA levels were significantly associated with adverse clinical features, including tumor stage, metastatic burden, and relapse risk. Importantly, elevated global miRNA levels were observed prior to clinically detectable disease progression, suggesting their potential role as early indicators of recurrence. This global approach contrasts with single-marker strategies and indicates that the overall burden of circulating miRNAs may itself carry prognostic information (145).

**Table 3:** Circulating microRNAs as non-invasive biomarkers for early detection, risk prediction, and prognostic stratification in breast cancer.

miRNA	Sample type	Clinical application	Key finding	Population/Setting	Reference
miR-21-3p, miR-21-5p, miR-99a-5p	Serum	Diagnosis	Three-miRNA panel with high diagnostic accuracy distinguishing BC patients from controls	Breast cancer patients vs healthy individuals	(140)
miR-155	Serum	Diagnosis/Monitoring	Elevated in BC; decreases after surgery/chemotherapy indicating treatment response	Breast cancer patients	(141)
miR-424, miR-423, miR-660, let-7i	Urine (exosomal)	Diagnosis	High sensitivity and specificity for non-invasive detection	Breast cancer patients vs healthy controls	(142)
miR-373	Circulating (serum/plasma)	Diagnosis/Prognosis	Upregulated; associated with VEGF and cyclin D1 indicating angiogenesis and proliferation	Breast cancer patients	(143)
miR-513a-5p	Leukocytes	Risk Prediction	Upregulated years before diagnosis; associated with hormonal factors	Prospective cohort (pre-diagnostic samples)	(144)
Global cf-miRNA levels	Plasma/serum	Prognosis/Recurrence	Higher total levels associated with tumor stage, metastasis, and relapse risk	Large clinical cohort	(145)
Multiple miRNAs (integrative signature)	Tumor + circulating	Prognosis	Associated with distant relapse-free survival and gene regulatory networks	Integrative molecular analysis	(146)
miR-9	Circulating/tissue	Prognosis	Associated with EMT, stemness, high grade tumors, poor survival	Breast cancer patients	(147

BC, breast cancer; VEGF, vascular endothelial growth factor; EMT, epithelial–mesenchymal transition.

At a more mechanistic level, integrative analyses have further elucidated the prognostic significance of specific miRNAs. Buffa et al. (146) applied a combined miRNA–mRNA profiling strategy to identify miRNAs independently associated with distant relapse-free survival. Their findings demonstrated that prognostic miRNAs were not merely correlated with clinical outcomes but were also functionally linked to regulatory gene networks involved in tumor progression. This systems-level perspective reinforces the concept that clinically informative miRNAs may reflect underlying biological processes rather than acting as isolated biomarkers. Additionally, specific miRNAs have been associated with aggressive tumor phenotypes and poor clinical outcomes. Gwak et al. (147) reported that elevated miR-9 expression was strongly associated with EMT, breast cancer stem cell phenotypes, and unfavorable clinicopathologic features, including higher tumor grade and proliferation index. Furthermore, increased miR-9 levels were identified as an independent predictor of reduced disease-free survival, highlighting its potential utility as a marker of tumor aggressiveness. These findings underscore the importance of miRNA profiling in identifying high-risk patient subgroups that may benefit from more intensive therapeutic strategies. Collectively, these clinical studies illustrate the multifaceted role of circulating miRNAs in breast cancer. From early detection and long-term risk assessment to prognostic stratification and identification of aggressive disease phenotypes, miRNAs offer a versatile and biologically grounded biomarker platform. However, despite these promising findings, several challenges remain, including variability in sample sources, analytical methods, and normalization strategies across studies. Therefore, while the integration of circulating miRNAs into clinical practice appears increasingly feasible, further standardization and large-scale validation are required to fully realize their translational potential.

## 10 Exercise-induced systemic immune modulation in breast cancer: integrating tumor microenvironment dynamics and therapeutic implications

Growing evidence suggests that the biological effects of exercise in breast cancer extend beyond localized molecular alterations and involve systemic immune reprogramming that may influence tumor behavior. Rather than acting through a single pathway, exercise appears to orchestrate a dynamic interplay between circulating immune components and the tumor microenvironment (TME), thereby shaping antitumor immunity in a context-dependent manner. Two complementary conceptual frameworks have been proposed: one emphasizes exercise-induced modulation of host immune surveillance, while the other highlights the endocrine-like effects of skeletal muscle–derived factors, known as myokines, which may indirectly influence tumor progression (148).

At the systemic level, acute and chronic exercise interventions have been shown to alter the distribution and functional state of immune cell populations relevant to tumor control. Transient mobilization of cytotoxic immune cells, including CD8^+^ T cells and NK cells, has been consistently observed following moderate-intensity exercise, accompanied by reductions in immunosuppressive cell subsets such as myeloid-derived suppressor cells (MDSCs) (149). These shifts suggest a temporary rebalancing of circulating immune profiles toward a more anti-tumorigenic phenotype. However, the magnitude and persistence of these effects appear to depend on both exercise intensity and disease characteristics, indicating that immune mobilization is not uniformly beneficial across all contexts.

Beyond transient immune cell redistribution, exercise training may induce more sustained remodeling of the tumor immune microenvironment. Preclinical models have demonstrated that structured exercise can enhance intratumoral infiltration of CD8^+^ T cells and improve their effector function, partly mediated through chemokine signaling pathways such as CXCL9/CXCL11–CXCR3 (150). Importantly, depletion of CD8^+^ T cells abolishes the antitumor effects of exercise in these models, suggesting a causal role for adaptive immunity in mediating exercise-associated tumor control. In parallel, exercise has been associated with vascular normalization, reduced hypoxia, and improved perfusion within tumors, which may further facilitate immune cell infiltration and function (150). The interaction between exercise and systemic inflammatory signaling also represents a key regulatory axis. Clinical and interventional studies indicate that exercise can modulate circulating cytokine profiles, although findings remain heterogeneous. Moderate physical activity during chemotherapy has been associated with shifts in cytokine patterns suggestive of enhanced immune responsiveness, including alterations in interleukins and chemokines involved in T helper cell polarization (151). Similarly, resistance training interventions in breast cancer survivors have demonstrated reductions in pro-inflammatory signaling within immune cell subsets, even in the absence of significant changes in systemic cytokine concentrations (152). These observations imply that exercise may exert immunomodulatory effects at both systemic and cellular levels, potentially decoupling circulating biomarkers from functional immune outcomes. An additional layer of complexity arises from the dose-dependent nature of exercise-induced immune modulation. Experimental evidence indicates that exercise intensity and duration critically determine whether exercise exerts beneficial or detrimental effects on tumor progression.

While moderate exercise appears to enhance antitumor immunity and delay tumor growth, excessively intense or prolonged exercise may promote metastatic dissemination and impair survival, potentially through dysregulation of immune cell ratios such as the neutrophil-to-lymphocyte ratio (153). This non-linear relationship underscores the importance of optimizing exercise prescriptions in oncological settings, as both insufficient and excessive stimuli may fail to achieve desired therapeutic effects. Importantly, systemic immune modulation induced by exercise may also interact with therapeutic responses. Preclinical data suggest that exercise can sensitize tumors to immune checkpoint blockade by enhancing T-cell–mediated antitumor activity and remodeling the immune microenvironment (154). However, clinical evidence remains limited and sometimes inconclusive. For instance, exploratory analyses of exercise interventions during neoadjuvant chemotherapy have shown only marginal or non-significant improvements in pathological response and tumor-infiltrating lymphocytes (TILs), highlighting the need for larger and more rigorously designed trials (155). These discrepancies may reflect differences in study design, patient populations, and exercise protocols, as well as the inherent complexity of immune–tumor interactions. Finally, large-scale computational analyses further support the central role of immune infiltration patterns in determining breast cancer outcomes and therapeutic responsiveness. Distinct immune phenotypes characterized by varying levels of immune cell infiltration have been associated with divergent prognostic trajectories and differential sensitivity to immunotherapy (156). These findings provide a broader systems-level context within which exercise-induced immune modulation may operate, suggesting that the impact of exercise could be contingent upon baseline immune landscape and tumor subtype. Taken together, current evidence supports a model in which exercise acts as a systemic regulator of immune–tumor crosstalk, influencing both circulating immune dynamics and the tumor microenvironment. However, these effects are highly context-dependent and modulated by exercise dose, tumor characteristics, and treatment conditions. Therefore, while exercise holds promise as an adjunct strategy for enhancing antitumor immunity, further mechanistic and clinical studies are required to define optimal intervention parameters and identify patient subgroups most likely to benefit.

## 11 Limitations and future perspectives

Despite the expanding interest in miRNAs as mediators linking exercise biology and breast cancer, the current body of evidence remains constrained by several conceptual and methodological limitations that hinder clear interpretation and clinical translation. While previous sections have outlined mechanistic insights and emerging clinical relevance, a critical evaluation reveals substantial gaps that must be addressed to advance the field. A major limitation lies in the pronounced heterogeneity of exercise interventions across studies. Existing research encompasses a wide spectrum of protocols, including aerobic, endurance, resistance, and high-intensity interval training, often differing in duration, intensity, and timing relative to cancer treatment. These variations are rarely standardized, making cross-study comparisons challenging and limiting the ability to define optimal exercise regimens for modulating miRNA profiles or improving clinical outcomes (70, 137). Importantly, some findings suggest that exercise intensity may exert divergent effects on tumor biology and immune responses, indicating that the relationship between exercise dose and molecular adaptation is not linear. This underscores the need for more precisely controlled intervention designs. Another critical issue is the variability in biological sample sources and analytical methodologies used to assess miRNAs. Studies have investigated miRNAs in tumor tissue, plasma, serum, extracellular vesicles, and even alternative biofluids, each reflecting different aspects of tumor biology and systemic physiology. However, inconsistencies in sample collection, RNA extraction, normalization strategies, and detection platforms contribute to substantial variability in reported results. This lack of methodological harmonization reduces reproducibility and complicates efforts to validate miRNAs as reliable biomarkers. Without standardized workflows, the translation of promising findings into clinical practice remains uncertain. A further limitation emerges from the disconnect between mechanistic and clinical evidence. Much of the current understanding of miRNA-mediated regulation—particularly in relation to angiogenesis, immune modulation, and metabolic reprogramming—derives from preclinical models. While these studies provide valuable mechanistic frameworks, their applicability to human breast cancer is not always straightforward. In contrast, clinical studies are often limited by small sample sizes, heterogeneous patient populations, and observational designs, which restrict causal inference and generalizability (137, 138). As a result, the integration of mechanistic insights with clinically meaningful outcomes remains incomplete. The intrinsic complexity of miRNA biology also presents a significant challenge. Individual miRNAs can target multiple genes and participate in interconnected signaling networks, including pathways such as PI3K/Akt, Wnt/β-catenin, NF-κB, and TGF-β. This pleiotropic nature means that the functional role of a given miRNA is highly context-dependent and may vary across tumor subtypes, disease stages, and microenvironmental conditions. Consequently, distinguishing between causal regulators and secondary markers becomes difficult, particularly in systemic contexts where exercise-induced changes may influence multiple pathways simultaneously.

Although circulating and exosomal miRNAs have demonstrated potential as minimally invasive biomarkers, their clinical utility is still limited by insufficient validation. Several studies have identified associations between specific miRNA signatures and diagnostic or prognostic parameters, yet these findings are often derived from relatively small or heterogeneous cohorts (140, 145). Moreover, discrepancies between tissue-derived and circulating miRNA profiles suggest that selective release mechanisms may influence biomarker interpretation. This adds another layer of complexity, as circulating miRNAs may not always directly reflect tumor-intrinsic processes. Looking forward, several key directions can be identified to address these limitations. First, there is a clear need for standardized exercise intervention protocols that account for intensity, duration, and patient-specific factors. Establishing consensus guidelines would facilitate comparability across studies and help identify optimal exercise strategies for modulating tumor biology. Second, methodological harmonization in miRNA analysis including standardized sample processing and normalization techniques, is essential to improve reproducibility and enable large-scale validation. In addition, future research should prioritize integrative, multi-omics approaches to better capture the complexity of miRNA-mediated regulation. Combining miRNA profiling with transcriptomic, proteomic, and metabolomic data may provide a more comprehensive understanding of the molecular networks influenced by exercise and cancer progression. Such approaches could help distinguish functional drivers from secondary effects and identify more robust biomarker signatures. Importantly, well-designed longitudinal and interventional clinical studies are needed to establish causal relationships between exercise-induced systemic changes and tumor-specific molecular responses. Incorporating mechanistic endpoints will be critical for bridging the gap between experimental and clinical evidence. Future intervention studies should also investigate the translational relevance of exercise-responsive miRNAs identified in murine tumor models and evaluate their applicability as predictive and prognostic biomarkers in clinical breast cancer settings. Greater attention to the molecular signaling pathways regulated by exercise-induced miRNAs may improve understanding of tumor biology and support the development of personalized intervention strategies tailored to individual patient responses to exercise. Integrating miRNA profiling with exercise-based therapeutic approaches may further contribute to precision oncology and individualized patient management. Finally, the concept of personalized exercise oncology represents a promising future direction. Given the variability in miRNA responses and tumor behavior, tailoring exercise interventions based on individual molecular and clinical characteristics may enhance therapeutic efficacy. In this context, miRNAs could serve not only as biomarkers but also as tools for guiding and monitoring personalized interventions. In summary, while current evidence supports a meaningful role for miRNAs in mediating interactions between exercise and breast cancer biology, significant methodological and conceptual challenges remain. Addressing these limitations through standardized, integrative, and clinically oriented research will be essential to fully realize the translational potential of miRNA-based strategies in breast cancer management.

## 12 Conclusion

A comprehensive integration of molecular, systemic, and inter-organ signaling perspectives suggests that miRNAs may play a more multifaceted role in breast cancer biology than previously appreciated. Rather than being confined to intracellular regulatory functions or serving solely as passive biomarkers, miRNAs appear to participate in broader regulatory networks that potentially link tumor-intrinsic signaling with systemic physiological adaptations. This interconnected view becomes particularly relevant when considering exercise as a systemic stimulus capable of modulating both circulating and tissue-associated miRNA profiles. The collective evidence synthesized in this review indicates that miRNAs are consistently associated with key oncogenic and tumor-regulatory processes, including proliferation, metastasis, angiogenesis, immune modulation, and metabolic adaptation. Their capacity to influence multiple signaling pathways suggests that they may function as integrative regulators within complex biological systems. Importantly, these effects are not restricted to the tumor microenvironment but may extend to systemic communication networks involving skeletal muscle, circulating factors, and extracellular vesicles. Such observations support the notion that breast cancer progression may be shaped by continuous interactions between tumor cells and host physiology, rather than by isolated molecular events. Within this broader framework, exercise may be conceptualized as a modulator of systemic signaling that could influence tumor-related processes through miRNA-dependent mechanisms. Exercise-induced alterations in circulating miRNAs, muscle-derived signaling molecules, and immune responses suggest a potential capacity to reshape the tumor microenvironment in a manner that may be less conducive to tumor progression. At the same time, tumor-derived signals, including extracellular vesicle-associated miRNAs, may exert reciprocal effects on distant tissues such as skeletal muscle, further reinforcing the concept of a bidirectional communication axis. These observations imply that tumor progression and systemic adaptation are likely governed by a dynamic balance of regulatory signals. An important implication of this synthesis is that circulating and extracellular vesicle-associated miRNAs may act as intermediaries that reflect, and possibly contribute to, the translation of systemic physiological changes into tumor-relevant molecular responses. While this perspective highlights their potential functional relevance, it also underscores the complexity of interpreting miRNA signatures, as their biological effects appear to be highly context-dependent and influenced by tumor subtype, disease stage, and host-related factors such as metabolic and inflammatory status. From a conceptual standpoint, this review proposes that miRNAs may extend beyond their conventional role as biomarkers and could be considered components of a broader systemic regulatory network linking exercise-induced adaptations to tumor biology.

By bringing together evidence from molecular signaling, circulating biomarkers, and muscle–tumor communication pathways, a multi-layered framework is suggested in which miRNAs may contribute to coordinating interactions between local and systemic processes. Although this framework remains to be fully validated, it provides a basis for re-examining the role of miRNAs within the context of exercise oncology. Looking ahead, further progress in this field will likely depend on integrative research approaches that bridge molecular mechanisms with clinically relevant outcomes. In particular, clarifying how exercise-induced changes in circulating and tissue-specific miRNAs relate to tumor behavior, treatment response, and long-term prognosis will be essential. Such efforts may benefit from combining multi-level analyses with carefully designed clinical studies that account for variability in both biological and intervention-related factors. Furthermore, future intervention studies integrating exercise oncology with molecular profiling may help clarify the translational relevance of miRNAs in breast cancer management. Preclinical evidence obtained from murine tumor models has already demonstrated the potential utility of circulating and tissue-associated miRNAs as biomarkers reflecting tumor biology, systemic adaptation, and responsiveness to exercise-related stimuli. Expanding these approaches into clinically oriented investigations may improve understanding of how miRNA-regulated signaling pathways contribute to inter-individual variability in treatment response and exercise adaptation. Such insights could ultimately support the development of more personalized intervention strategies based on molecular and physiological profiling of patients with breast cancer. In summary, miRNAs appear to occupy a central and potentially integrative position within the complex interplay between breast cancer and host physiology. Their involvement in both tumor-associated signaling and systemic adaptation suggests that they may represent a valuable link between molecular mechanisms and whole-body responses. Continued investigation of these interactions may contribute to a more nuanced understanding of breast cancer biology and support the development of more targeted and personalized intervention strategies.
